# Therapeutic potential of BH3-mimetics and NK cell-mediated immunotherapy in T-ALL

**DOI:** 10.1038/s41419-026-08698-x

**Published:** 2026-04-04

**Authors:** Colin Fortner, Alexandra Niedermayer, Melina Maria Bäuerle, Maren Christiane Wichert, Christian Jörg Braun, Klaus-Michael Debatin, Meike Vogler, Lüder Hinrich Meyer, Felix Seyfried

**Affiliations:** 1 Department of Pediatrics and Adolescent Medicine, Ulm University Medical Center, Ulm, Germany; 2 International Graduate School in Molecular Medicine, Ulm University, Ulm, Germany; 3 German Center for Child and Adolescent Health (DZKJ), partner site Ulm, Ulm, Germany; 4 Department of Pediatrics, Dr. von Hauner Children’s Hospital, University Hospital, LMU Munich, Munich, Germany; 5 Department of Pediatrics, Goethe University Frankfurt, Frankfurt, Germany; 6 Institute for Experimental Pediatric Hematology and Oncology, Goethe University Frankfurt, Frankfurt, Germany; 7 Present Address: Department of Pediatric Hematology, Oncology and Stem Cell Transplantation, University Medical Center Regensburg, Regensburg, Germany

**Keywords:** Acute lymphocytic leukaemia, Apoptosis

## Abstract

T-cell acute lymphoblastic leukemia (T-ALL) is an aggressive malignancy of T-cell precursors. Although the survival rates have improved with the use of intensive chemotherapy, the emergence of relapse as well as treatment-related morbidity and mortality remain major challenges. Novel treatment approaches include the inhibition of anti-apoptotic regulators or cellular immunotherapies. Here, we analyzed the sensitivity of T-ALL to inhibitors of BCL-2 (venetoclax), BCL-XL (A1331852), MCL-1 (AZD5991) and dual inhibition of BCL-2/BCL-XL (AZD4320) and evaluated their combination effects with natural killer (NK) cells. While only early T-cell precursor (ETP) ALL was sensitive to BCL-2 inhibition, MCL-1 inhibition alone was not effective in most cell lines and patient-derived xenograft (PDX) samples. For BCL-XL and dual BCL-2/BCL-XL inhibition, we observed heterogeneous sensitivities, which were associated with anti-apoptotic dependencies on the respective BCL-2 family members as assessed by BH3-profiling. Moreover, we identified functional shifts in anti-apoptotic dependencies upon exposure to AZD4320 or AZD5991 alone and synergistic effects when both inhibitors were combined with each other, allowing cell death induction in resistant samples. We then explored the potential use of apoptosis-inducing drugs as sensitizers for immunotherapy. Therefore, we investigated the potential of NK cell-mediated killing in T-ALL and found heterogeneous sensitivity, with some cell lines showing responses even at low effector-to-target (E:T) ratios. Importantly, NK cell-mediated killing could be further enhanced by combining NK cells with AZD4320, proposing this combination as a potential effective treatment. Taken together, we demonstrated promising potential of BH3-mimetics and NK cells for the treatment of T-ALL alone and in combination, warranting further preclinical and potential clinical evaluation.

## Introduction

T-cell acute lymphoblastic leukemia (T-ALL) arises from the malignant transformation of T-cell progenitors [1, 2]. It is less common than B-cell precursor (BCP) ALL, accounting for approximately 25% of adult and 15% of pediatric ALL cases [3–5]. T-ALL subclasses can be categorized based on the stage of T-cell maturation or molecular features [6–8]. Early T-cell precursor (ETP) ALL is a high-risk subtype derived from immature T-cell progenitors, which is characterized by a distinct gene expression and surface marker profile and is associated with a poor prognosis [7–11]. Although outcomes of patients with de-novo T-ALL have improved with the use of multi-agent chemotherapy regimens, they remain poor in patients who have relapsed. In addition, the use of intensive chemotherapy is associated with high rates of treatment-related morbidity and mortality, highlighting the need for novel therapeutic strategies [12, 13].

One potential treatment approach is to target the intrinsic apoptosis pathway, which is frequently dysregulated in leukemias [14, 15]. This pathway is controlled by pro- and anti-apoptotic B-cell lymphoma 2 (BCL-2) family proteins, which maintain the balance between cell survival and cell death in non-malignant cells [16, 17]. To target anti-apoptotic BCL-2 family proteins, BCL-2 homology 3 (BH3)-mimetics were developed, which functionally mimic sensitizer proteins by binding to and antagonizing anti-apoptotic BCL-2 family members [18, 19]. Venetoclax, a selective BCL-2 inhibitor, is the first BH3-mimetic to receive clinical approval, demonstrating efficacy particularly in chronic lymphocytic leukemia (CLL) and acute myeloid leukemia (AML) [20–23]. However, insensitivity and resistance to venetoclax have been described, for example by upregulation of other anti-apoptotic BCL-2 family members [24–26]. Therefore, other inhibitors targeting MCL-1 (e.g., S63845, AZD5991) or BCL-XL (e.g., A1331852) are potential drug candidates, but are associated with cardiotoxicity [27–29] or severe thrombocytopenia, respectively [30, 31]. In addition, combined inhibitors of BCL-2 and BCL-XL have been developed. For example, navitoclax has been investigated in clinical trials, but has been associated with dose-limiting thrombocytopenia [32, 33]. AZD4320, another BCL-2/BCL-XL inhibitor, has shown anti-tumor effects in different cancer models with manageable thrombocytopenia [34].

In addition to BH3-mimetics, immunotherapies are emerging as a novel therapeutic option for T-ALL [35, 36]. Natural killer (NK) cell-based therapies are a promising approach with a favorable safety profile, avoiding graft-versus-host disease and cytokine release syndrome [37, 38]. Moreover, NK cells can be used “off-the-shelf” in an allogeneic setting [39, 40]. Interestingly, NK cell-mediated killing has been described to involve the intrinsic apoptosis pathway. Moreover, recent publications have shown that combining NK cells together with BH3-mimetics results in enhanced tumor cell killing in preclinical cancer models [41, 42].

In this study, we assessed the sensitivity of T-ALL cell lines and patient-derived xenograft (PDX) samples for the BH3-mimetics venetoclax (BCL-2), A1331852 (BCL-XL), AZD4320 (BCL-2/BCL-XL) and AZD5991 (MCL-1), searched for markers of response and assessed combination effects between different BH3-mimetics and between BH3-mimetics and NK cells.

## Materials and methods

### T-ALL cell lines

Loucy, ALL-SIL, MOLT-4, BE-13, CCRF-CEM and Jurkat cells were purchased from DSMZ, Germany. All cell lines were cultured in RPMI-1640 medium supplemented with 20% fetal bovine serum, 1% Penicillin/Streptomycin and 1% L-Glutamine at 5% CO_2_ and 37°C. All cell lines were authenticated by short tandem repeat (STR) profiling (GenePrint 10 System, Promega) and tested negative for mycoplasma (MycoAlert Mycoplasma Detection Kit, Lonza).

### T-ALL patient-derived xenograft samples

Primary leukemia samples of T-ALL patients were collected after written informed consent in accordance with our institution’s ethical review board. Patient-derived xenograft (PDX) samples were generated by intravenous transplantation of cells into female NSG (NOD.Cg-*Prkdc*^*scid*^ *Il2rg*^*tm1Wjl*^/SzJ) or NOD/SCID (NOD.CB17-Prkdcscid) mice as described before [43]. Animal experiments were approved by the appropriate authority (Regierungspräsidium Tübingen). For experiments, PDX cells were cultured in AIM-V medium (Gibco) without additional supplements.

### Statistical analysis

Statistical analyses were conducted using Microsoft Excel and GraphPad Prism software. Combination effects and synergy scores were determined using SynergyFinder and the bliss independence model [44–46].

Additional materials and methods are provided in the supplementary data. Full length uncropped western blots are presented in the supplemental material.

## Results

### Characterizing the sensitivity of T-ALL cell lines to BH3-mimetics

**Fig. 1:**
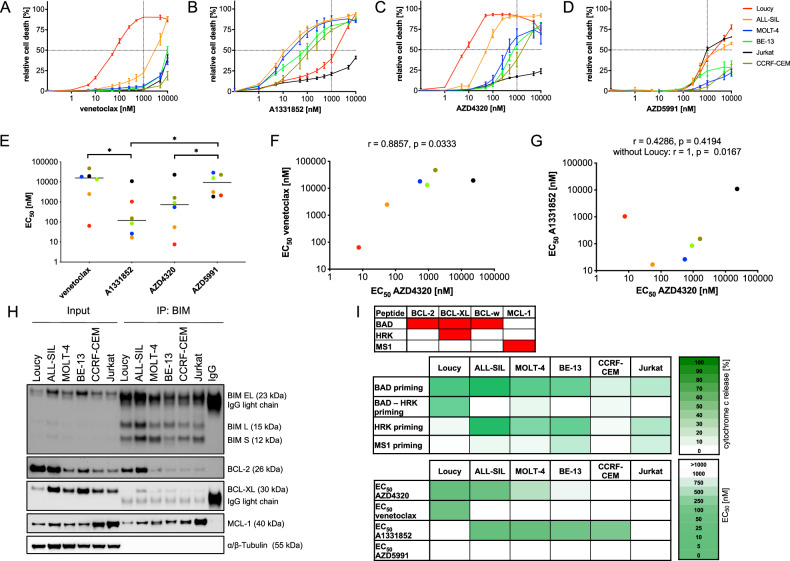
BH3-profiling predicts sensitivity of T-ALL cell to BH3-mimetics. T-ALL cell lines were exposed for 48 h to increasing concentrations (0.1, 1, 5, 10, 50, 100, 250, 500, 1000, 5000, 10,000 nM) of **A** venetoclax, **B** A1331852 **C** AZD4320 or **D** AZD5991 before analysis of cell death by propidium iodide staining and flow cytometry. Data points indicate mean values of *N* = 3 independent experiments in triplicates and error bars standard deviation. **E** Scatter plot of EC_50_ values of the four BH3-mimetics with individual data points of all cell lines and medians (lines). Mann-Whitney test; * indicates significance, *p* < 0.05. **F**, **G** Association of the EC_50_ values of AZD4320 with those of venetoclax and A1331852 in all cell lines. Spearman correlation (two-tailed); r, correlation coefficient; p, significance. **H** Protein extracts of T-ALL cell lines were co-incubated with anti-BIM-antibody overnight, co-immunoprecipitation and input protein extracts were subjected to western blot. Jurkat was used for IgG isotype control. Basal levels of proteins and protein complexes with BIM are shown in six T-ALL cell lines (representative of *N* = 3). **I** For BH3-profiling, cell lines were incubated with BH3-peptides before fixing and cytochrome c staining. Cytochrome c release after peptide exposure was analyzed by flow cytometry and values were normalized to alamethicin as positive control and DMSO as negative control. Means of *N* = 3 independent experiments in triplicates. BAD–HRK is the calculation of BCL-2 dependency by subtracting HRK priming (which targets only BCL-XL) from BAD priming (which targets both BCL-2 and BCL-XL). The lower heatmap shows the EC_50_ values of the inhibitors indicated.

We first tested the sensitivity of six T-ALL cell lines to inhibition of anti-apoptotic BCL-2 family members. We exposed all cell lines to increasing concentrations of selective inhibitors of BCL-2 (venetoclax), BCL-XL (A1331852) and MCL-1 (AZD5991) and to the dual BCL-2/BCL-XL inhibitor AZD4320 and determined cell death rates by flow cytometry and propidium iodide staining (Supplementary Fig. 1A–C). For inhibition of BCL-2, we found insensitivity (EC_50_ > 1000 nM) in all cell lines except for the ETP cell line Loucy (Fig. 1A). Of note, BCL-2 dependency has previously been described as a feature of ETP-ALL [24, 47]. For selective inhibition of BCL-XL, we found heterogeneous sensitivity, with four cell lines being sensitive and two being more resistant (Fig. 1B). Interestingly, when the effects of selective BCL-XL inhibition were compared with those of combined BCL-2/BCL-XL inhibition (Fig. 1C), all cell lines except for the BCL-2-dependent line Loucy, were more sensitive to the selective BCL-XL inhibitor, suggesting that the dual inhibitor is more active in BCL-2-dependent than in BCL-XL-dependent cells. Finally, we found that all T-ALL cell lines were insensitive for MCL-1 inhibition (Fig. 1D). Overall, we found that A1331852 was more effective than both venetoclax and AZD5991, while AZD4320 was more effective than AZD5991 (Fig. 1E). Furthermore, we found an association of the sensitivities of the dual BCL-2/BCL-XL inhibitor AZD4320 with those of BCL-2-selective venetoclax (Fig. 1F). In addition, when analyzing only typical T-ALL without the ETP-ALL cell line Loucy, we found an association of the sensitivities of AZD4320 with those of the BCL-XL inhibitor A1331852 (Fig. 1G).

To confirm these data and demonstrate that apoptosis is the mechanism of cell death induced by the BH3-mimetics in T-ALL, we next performed Annexin V/PI staining and Caspase-Glo 3/7 assays in three representative cell lines (Loucy, MOLT-4 and Jurkat). Annexin V/PI staining showed that sensitive cell lines became double-positive after 48 h (Supplementary Fig. 2A–C), indicating late-stage apoptosis [48]. In addition, caspase-3/7 activity, another marker of apoptosis, was increased upon exposure to BH3-mimetics (Supplementary Fig. 2D). Of note, while caspase activity increased at the same concentrations at which cell death was observed by both PI and Annexin V staining (Fig. 1A–D, Supplementary Fig. 2C), the intensity of caspase activation did not correlate with the extent of cell death induction, as Jurkat showed the highest caspase fold change in response to BCL-XL inhibition despite being the least sensitive.

Taken together, our data suggest that T-ALL shows heterogeneous sensitivity to BCL-XL inhibition, while ETP-ALL may be particularly sensitive to BCL-2 inhibition, consistent with previously reported findings [24, 47].

### Functional parameters of apoptosis signaling predict sensitivity to BH3-mimetics

Next, we analyzed whether T-ALL sensitivity to BH3-mimetics was associated with the levels of anti-apoptotic BCL-2 family proteins or their complexes with BIM, as potential markers of response. Co-immunoprecipitation of BIM, followed by Western blotting, revealed heterogeneous expression levels of these apoptosis regulators across cell lines (Fig. 1H, Supplementary Fig. 3A). We performed densitometric quantification of BCL-2, BCL-XL and MCL-1 (input), and BIM-bound fractions (IP:BIM) across three independent experiments and correlated the mean values with the EC_50_ values for each BH3-mimetics (Table 1). BCL-2 expression (input) significantly correlated with venetoclax sensitivity, while A1331852 sensitivity correlated with BIM-bound BCL-XL (IP:BIM). No significant correlation was observed between AZD5991 EC_50_ values and MCL-1 levels. To further integrate anti-apoptotic dependencies, we applied the “mediators of apoptosis combinatorial” (MAC) score [49]. The MAC score was developed based on the hypothesis that sensitivity to drugs that target the BCL-2 family is not determined by the expression of the target protein alone, but rather by the interaction between multiple BCL-2 family members. The ratio of BCL-2 to BCL-XL and MCL-1 significantly correlated with EC_50_ values of venetoclax and AZD4320 (Table 1). However, the ratios did not correlate with A1331852 and AZD5991 sensitivity. To verify the specificity of the anti-BIM antibody used for immunoprecipitation, we included parallel IgG controls for all cell lines, which generated no specific protein signals. Additionally, to exclude interference from IgG light chains, we confirmed the key findings using heavy-chain-specific fluorescent secondary antibodies (Supplementary Fig. 3B). Overall, our data indicate that sensitivity or resistance to these inhibitors is partly reflected in protein levels and ratios, but not in all cases. Next, we employed BH3-profiling to assess the functional dependence of leukemia samples on anti-apoptotic BCL-2 family proteins (Fig. 1I). This involved measuring cytochrome c release in response to pro-apoptotic BH3-peptides targeting these proteins (Supplementary Fig. 4A, B). Importantly, BH3-profiling results were closely associated with the observed sensitivities to the inhibitors, indicating that BH3-profiling could serve as a reliable tool to predict the sensitivity of T-ALL to apoptosis-inducing drugs.

**Table 1:** Association of BH3-mimetic sensitivities with protein levels.

(a)	Compounds	Venetoclax		A1331852		AZD4320		AZD5991	
	Protein levels	*r*_s_	*p*	*r*_s_	*p*	*r*_s_	*p*	*r*_s_	*p*
**Input**	BCL-2	−0.943	0.017*	−0.371	0.497	−0.943	0.017*	−0.657	0.175
	BCL-XL	0.143	0.803	−0.429	0.419	0.314	0.564	0.314	0.564
	MCL-1	−0.143	0.803	0.657	0.175	0.086	0.919	−0.257	0.658
**(b)**	**Protein ratios**	***r***_**s**_	***p***	***r***_**s**_	***p***	***r***_**s**_	***p***	***r***_**s**_	***p***
**Input**	BCL-2/BCL-XL	−0.886	0.033*	−0.429	0.419	−1.000	0.003**	−0.771	0.103
	BCL-2/MCL-1	−0.943	0.017*	−0.371	0.497	−0.943	0.017*	−0.657	0.175
	BCL-2/(BCL-XL + MCL-1)	−0.943	0.017*	−0.371	0.497	−0.943	0.017*	−0.657	0.175
	BCL-XL/MCL-1	0.086	0.919	−0.714	0.136	0.086	0.919	0.200	0.714
	BCL-XL/(BCL-2 + MCL-1)	0.429	0.419	−0.429	0.419	0.371	0.497	0.657	0.175
**(b)**	**Protein complexes**	***r***_**s**_	***p***	***r***_**s**_	***p***	***r***_**s**_	***p***	***r***_**s**_	***p***
**IP: BIM**	BCL-2-BIM	−0.714	0.136	−0.371	0.497	−0.943	0.017*	−0.714	0.136
	BCL-XL-BIM	−0.086	0.919	−0.886	0.033*	−0.086	0.919	−0.143	0.803
	MCL-1-BIM	0.714	0.136	0.371	0.497	0.943	0.017*	0.714	0.136

Densitometric analysis of protein levels, ratios and complexes was performed using ImageJ. Quantified protein expression of the western blots in Fig. 1H and Supplementary Fig. 3A was normalized to Tubulin for input samples and to BIM for immunoprecipitation (IP) samples. Mean values were then correlated with the EC_50_ values of the different BH3-mimetics (shown in Supplementary Fig. 1C). Spearman correlation (two-tailed); *r*_s_, correlation coefficient; *p*, significance.

*p < 0.05, **p < 0.01.

### Shifted anti-apoptotic dependencies upon exposure of T-ALL to AZD4320 and AZD5991

**Fig. 2:**
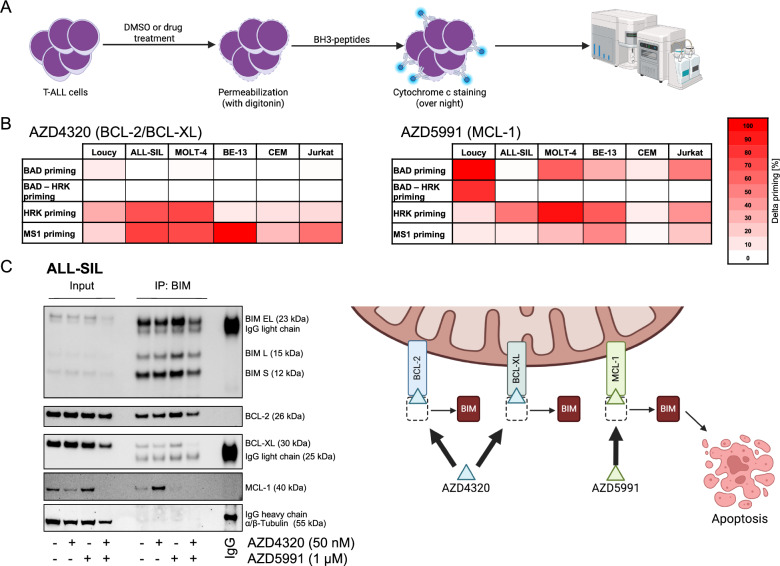
AZD4320 and AZD5991 induce shifts in anti-apoptotic dependencies. **A** Graphical scheme of dynamic BH3-profiling. Cell lines were exposed to drug or DMSO for 4 h (Loucy) or 2 h (other cell lines) before permeabilization and peptide exposure, fixation and cytochrome c staining. Cytochrome c release was analyzed using flow cytometry. Values were normalized to alamethicin as positive control and to DMSO as negative control. *N* = 3 independent experiments in triplicates. BAD–HRK is the calculation of BCL-2 dependency by subtracting HRK priming (which targets only BCL-XL) from BAD priming (which targets both BCL-2 and BCL-XL). Created with BioRender.com. **B** Delta-priming was determined after incubation with AZD4320 (left) and AZD5991 (right) and calculated by subtracting normalized cytochrome c release of DMSO-treated cells from normalized cytochrome c release of drug-treated cells. **C** ALL-SIL cells were treated with 50 nM AZD4320 and/or 1 µM AZD5991 for 4 h. After treatment, protein extraction was performed and protein lysates were subjected to co-immunoprecipitation with anti-BIM-antibody. Co-immunoprecipitation and input protein extracts were subjected to western blot analyses. Representative of *N* = 3 different cell lines. The graphical scheme illustrates the mechanism of action of the BH3-mimetics used for the treatment before co-immunoprecipitation. AZD4320 and AZD5991 bind to their target proteins, releasing BIM from them, which subsequently activates apoptosis. Created with BioRender.com.

Next, we analyzed changes in the apoptotic dependencies of T-ALL cell lines following exposure to AZD4320 and AZD5991 by using dynamic BH3-profiling (Fig. 2A). We observed that exposure of the cells to the dual BCL-2/BCL-XL inhibitor AZD4320 caused a shift towards increased MCL-1 dependence. On the other hand, exposure to the MCL-1 inhibitor AZD5991 resulted in a shift towards BCL-2 dependence in Loucy cells and towards BCL-XL in the other cell lines (Fig. 2B). To further elucidate these findings at the molecular level, we performed BIM immunoprecipitation and western blot after exposure to AZD4320 and AZD5991 alone and in combination (Fig. 2C, Supplementary Fig. 3C). As illustrated in the graphical scheme in Fig. 2C, AZD4320 and AZD5991 are assumed to act by binding to their respective targets and displacing pro-apoptotic proteins such as BIM, thereby promoting apoptosis. This provides a rationale for assessing shifts in anti-apoptotic proteins bound to BIM to gain mechanistic insight. We observed a marked increase in BIM-bound MCL-1 after AZD4320 treatment in Loucy and ALL-SIL. In BE-13, no direct increase in BIM-bound MCL-1 compared to DMSO was detectable. However, AZD4320 induced a pronounced reduction in total MCL-1 protein levels (input), indicating a relative increase in MCL-1 binding to BIM. Treatment with AZD5991 decreased MCL-1 binding to BIM in all cell lines. Combined inhibition resulted in lower levels of anti-apoptotic proteins bound to BIM compared to either single agent, and prevented the compensatory increase in MCL-1 binding observed with AZD4320 alone. Although mostly similar patterns were found across the three cell lines, some differences were apparent. Compensatory binding of BCL-2 and BCL-XL to BIM after AZD5991 exposure was most prominent in ALL-SIL, whereas Loucy showed compensatory binding mainly of BCL-2, and BE-13 did not exhibit clear compensatory binding following AZD5991 treatment.

### Synergism of combined inhibition of anti-apoptotic proteins

**Fig. 3:**
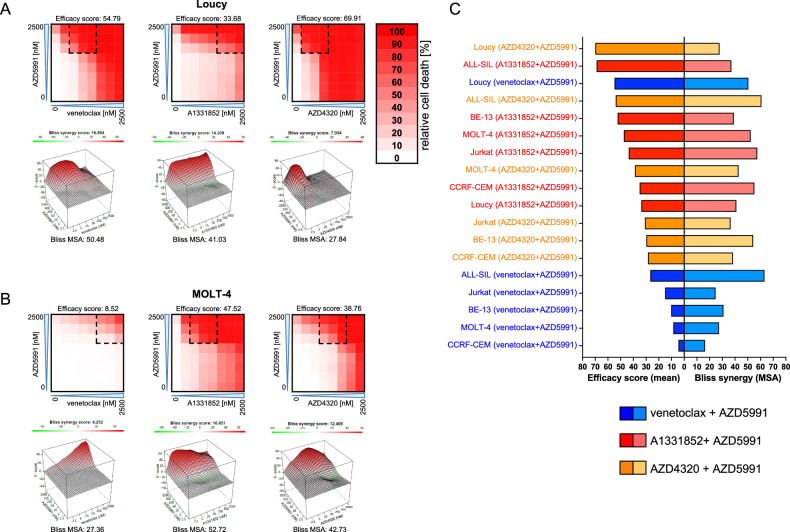
Synergistic cell death induction by AZD4320 and AZD5991 in T-ALL cell lines. Cell death was assessed upon exposure of T-ALL cell lines to increasing concentrations (2.5, 5, 25, 50, 250, 500, 2500 nM) of venetoclax, A1331852 or AZD4320 and/or AZD5991 for 48 h. The heatmaps show dose-response matrix analyses based on cell death assessed by propidium iodide (PI) staining in **A** Loucy and **B** MOLT-4. Mean values of triplicates are shown in the heatmaps. The dashed lines indicate the most synergistic areas. Efficacy scores were calculated as means of all normalized cell death rates across the matrix. Interaction landscapes of the combination effects are shown in the respective lower panels. Bliss synergy scores and most synergistic area (MSA) scores were calculated using SynergyFinder. Bliss synergy scores of less than −10 indicate antagonism, scores between −10 and 10 indicate additive effects and scores above 10 indicate synergism. **C** Efficacy scores and Bliss synergy scores of venetoclax, A1331852 and AZD4320 combinations with AZD5991 in T-ALL cell lines as shown in Fig. 3A, B and Supplemental Fig. 2A–D are shown, in order from highest to lowest efficacy score.

Based on these findings, we evaluated the combination effects of venetoclax, A1331852 or AZD4320 with the MCL-1 inhibitor AZD5991 by analyzing dose-response matrices in T-ALL cell lines using the Bliss independence model. This model calculates whether the observed combination response to two drugs is greater or lesser than the expected combination response in the absence of drug-drug interactions. Bliss synergy scores above 10 indicate synergism, scores between 10 and −10 indicate additive effects, and scores below −10 indicate antagonism [44–46]. Cell lines were exposed to increasing concentrations of two inhibitors for 48 h followed by PI staining to assess cell death rates. In the ETP-ALL cell line Loucy, we found particularly high activity when combining MCL-1 inhibition with inhibitors targeting BCL-2 (venetoclax, AZD4320) (Fig. 3A). In contrast, for the MOLT-4 cell line, we found high activity when combining MCL-1 inhibition with inhibitors targeting BCL-XL (A1331852, AZD4320) (Fig. 3B), with similar results observed in the other cell lines. (Supplementary Fig. 5A–D). Importantly, Bliss synergy scores above ten in the most synergistic area (MSA) were found across all cell lines, confirming robust synergy [44–46]. However, we observed that combinations of AZD5991 with venetoclax showed only low efficacy, except for Loucy, while the combinations with inhibitors of BCL-XL were more effective than those with venetoclax in all other cell lines (Fig. 3C).

To investigate why combination of AZD5991 with venetoclax was rather ineffective in T-ALL cell lines except Loucy, we performed BIM immunoprecipitation and western blot analysis after exposure to venetoclax and AZD5991 alone and in combination (Supplementary Fig. 6A–D). In all cell lines, venetoclax single treatment increased MCL-1 binding to BIM, and in Loucy it also increased BCL-XL binding to BIM. Interestingly, despite these changes, the reduction of BCL-2-bound BIM after venetoclax treatment was only modest. This may be explained by the short incubation time, which may not yet fully reflect the maximal effects of venetoclax, or by a redistribution of venetoclax to other pro-apoptotic binding partners besides BIM. Notably, combination treatment reduced binding of all three anti-apoptotic BCL-2 family members to BIM only Loucy, whereas ALL-SIL and BE-13 retained high levels of BIM-bound BCL-XL. This may explain why these cell lines survive venetoclax + AZD5991 treatment, while Loucy does not.

### Heterogeneous sensitivity and synergism of BH3 mimetics in T-ALL PDX samples

**Fig. 4:**
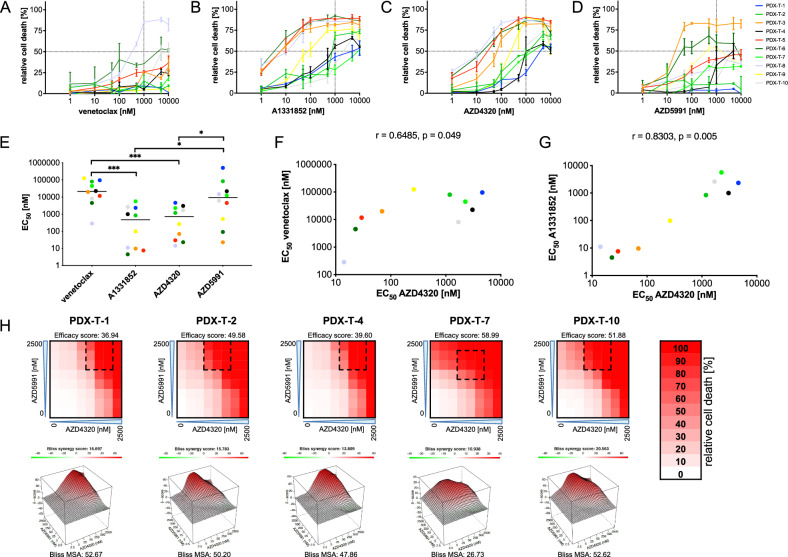
Efficacy of BH3-mimetics in T-ALL PDX-samples. T-ALL PDX-samples were exposed for 24 h to increasing concentrations (1, 10, 50, 100, 500, 1000, 5000, 10,000 nM) of **A** venetoclax, **B** A1331852 **C** AZD4320 or **D** AZD5991 before analysis of cell death by propidium iodide staining and flow cytometry. Experiments were performed in triplicates. Data points indicate mean values and error bars standard deviation. **E** Scatter plot of EC_50_ values of the four BH3-mimetics with individual data points of all PDX samples and medians (lines). Mann-Whitney test; p, significance, *p < 0.05, ***p < 0.001. **F**, **G** Association of the EC_50_ values of AZD4320 with those of venetoclax and A1331852 in PDX-samples. Spearman correlation (two-tailed); r, correlation coefficient; p, significance. **H** Five PDX-samples resistant to AZD4320 were exposed to increasing concentrations (2.5, 5, 25, 50, 250, 500, 2500 nM) of AZD4320 and/or AZD5991 for 24 h. Cell death was assessed by flow cytometry based on FSC/SSC criteria. Heatmaps (upper panels) show relative cell death rates in PDX samples. Mean values of triplicates are shown in the heatmaps. Efficacy scores were calculated as means of all normalized cell death rates across the matrix. Interaction landscapes of the combination effects are shown in the respective lower panels. Bliss synergy scores and most synergistic area (MSA) scores were calculated using SynergyFinder. Bliss synergy scores of less than -10 indicate antagonism, scores between −10 and 10 indicate additive effects and scores above 10 indicate synergism.

We next extended our analyses to primary T-ALL PDX samples. Most PDX samples were resistant to BCL-2 inhibition except for PDX-T-8, derived from an ETP (Fig. 4A, Supplementary Fig. 7). Heterogeneous sensitivities were found for BCL-XL inhibition, dual BCL-2/BCL-XL inhibition and MCL-1 inhibition (Fig. 4B–D, Supplementary Fig. 8A–C). Comparison of median sensitivities in PDX-samples showed that BCL-XL inhibitors were more effective in T-ALL than BCL-2 or MCL-1 inhibitors, with A1331852 and AZD4320 showing comparable efficacy (Fig. 4E). Interestingly, analysis of AZD4320 sensitivity revealed an association with venetoclax and a strong association with A1331852, highlighting the BCL-XL dependency in most samples and indicating that AZD4320 acts primarily through BCL-XL inhibition in T-ALL (Fig. 4F, G).

Lastly, we evaluated the combination effects of AZD4320 and AZD5991 in five PDX samples with low sensitivity to AZD4320 alone. Dose-response matrices confirmed synergistic activity in all samples with Bliss synergy scores above ten. Importantly, most synergistic effects were achieved at relatively low concentrations of AZD4320.

In summary, T-ALL PDX samples are largely sensitive to BCL-XL inhibition but mostly resistant to BCL-2 and MCL-1 inhibition, with some exceptions. Importantly, resistance to BCL-XL inhibition can be overcome using combination strategies.

Since different inhibitors of MCL-1 are currently investigated [29], we also evaluated the effects of another MCL-1 inhibitor. To this end, we tested the sensitivity of T-ALL cell lines and PDX samples to S63845, a specific MCL-1 inhibitor. Similar to AZD5991, we found that most T-ALL cell lines were insensitive to MCL-1 inhibition by S63845 (Supplementary Fig. 9A, B), while heterogeneous sensitivity was found in PDX samples (Supplementary Fig. 9C). We found a significant correlation between the EC_50_ values of AZD5991 and S63845 in PDX samples (Supplementary Fig. 9D). Furthermore, we investigated the combination effects of S63845 with other BH3-mimetics, identifying synergism and high efficacy in most samples, similar to AZD5991 (Supplementary Fig. 9E, F).

### T-ALL cells are sensitive to NK cell-mediated killing, which is enhanced by combining with AZD4320

**Fig. 5:**
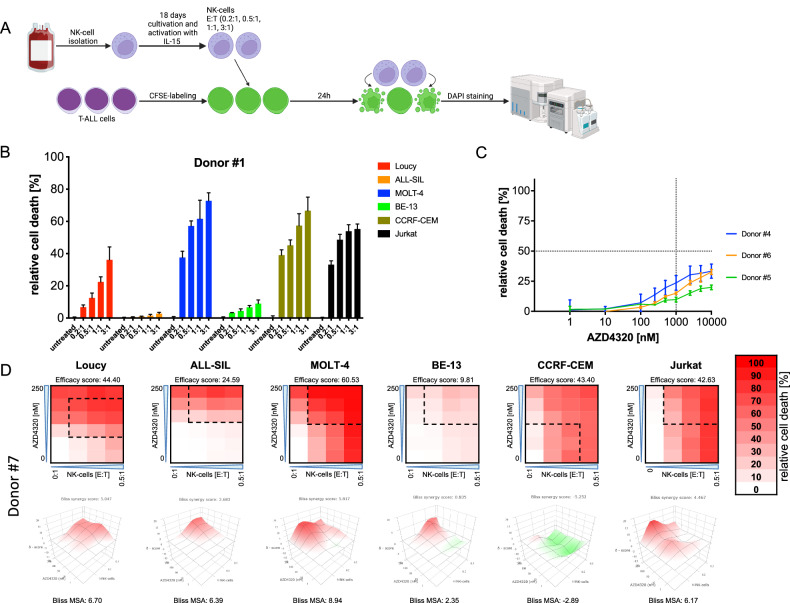
Sensitivity of T-ALL cell lines to NK cell-mediated killing and combinations of NK cells with AZD4320. **A** Graphical scheme of NK cell cytotoxicity assays. NK cells were isolated from buffy coats derived from healthy donors and amplified and activated in vitro for 18 days in medium containing IL-15. Before co-incubation, T-ALL cell lines were stained with CFSE to differentiate NK cells from leukemia cells using flow cytometry. After staining cell lines were incubated with increasing effector-to-target (E:T) -ratios (0.2:1, 0.5:1, 1:1 and 3:1) for 24 h before analysis of cell death by DAPI staining and flow cytometry. Created with BioRender.com. **B** Relative cell death rates of T-ALL cell lines are shown after incubation with NK cells for 24 h. Experiments were performed in triplicates using NK cells from *N* = 3 different donors, results from one representative donor are shown. Data points show mean values and error bars standard deviations. **C** NK cells derived from three different donors were incubated with increasing concentrations (1, 10, 100, 250, 500, 1000, 2500, 5000, 10,000 nM) of AZD4320 for 24 h before analysis of cell death by propidium iodide staining and flow cytometry. Data points indicate mean values and error bars standard deviation. **D** T-ALL cell lines were exposed for 24 h to increasing concentrations (1, 10, 50, 100, 250 nM) and/or increasing E:T-ratios of NK cells (0.1:1, 0.2:1, 0.5:1) before analysis of cell death by DAPI staining and flow cytometry. Heatmaps (upper panels) show relative cell death rates for one representative experiment in triplicates of *N* = 3 experiments with NK cells from different donors. Efficacy scores were calculated as means of all normalized cell death rates across the matrix. Interaction landscapes of the combination effects are shown in the respective lower panels. Bliss synergy scores and most synergistic area (MSA) scores were calculated using SynergyFinder. Bliss synergy scores of less than −10 indicate antagonism, scores between −10 and 10 indicate additive effects and scores above 10 indicate synergism.

An alternative strategy for treating high-risk leukemia is to activate immune cells for targeting and killing leukemia cells. Therefore, we sought to investigate whether BH3-mimetics can further enhance anti-leukemia effects of NK cells. We analyzed the sensitivity of T-ALL cell lines for NK cell-mediated killing using co-culture cytotoxicity assays (Fig. 5A, Supplementary Fig. 10A–C). Sensitivity varied, with three cell lines being sensitive, one being intermediate sensitive, and two lines being resistant (Fig. 5B). Notably, CCRF-CEM and Jurkat, which were relatively insensitive to BH3-mimetics, showed high sensitivity to NK cells, whereas Loucy and ALL-SIL, which were more sensitive to BH3-mimetics, showed low sensitivity to NK cell mediated killing. These data suggest that NK cell killing is independent of BH3-mimetic sensitivity. Additionally, sensitivity patterns were consistent across donors (Fig. 5B, Supplementary Fig. 11A), suggesting that NK cell-mediated killing of ALL cells is determined by leukemia-intrinsic factors rather than donor variability. Notably, when we analyzed gene expression patterns of T-ALL cell lines, we found that ALL-SIL and BE-13 cells lack *TRAILR1* and *TRAILR2* expression (Supplementary Fig. 11B). These receptors are known to play an important role in NK cell-mediated cell death [50]. Furthermore, when we compared the expression levels of activating and inhibitory NK cell receptor ligands [51, 52], we found that the intermediate-sensitive cell line Loucy expressed higher levels of multiple inhibitory ligands, especially MHC-I receptors, as compared to the sensitive cell lines. No clear pattern was observed for the expression of the activating ligands (Supplementary Fig. 11B).

Next, we asked how NK cell co-culture may alter the apoptotic dependencies in the leukemia cells. Interestingly, using BH3-profiling we found that exposure of T-ALL cells to NK cells resulted in changes in anti-apoptotic dependencies, particularly in increased BCL-XL-dependence (Supplementary Fig. 11C), providing a rational for combining NK cells with BH3-mimetics targeting BCL-XL.

When combining BH3-mimetics with NK cells, it is important to confirm that NK cells themselves are not sensitive to BH3-mimetics. We evaluated the sensitivity of IL-15 activated NK cells from three donors to AZD4320. All three donor-derived NK cells were resistant, with EC_50_ values above 10 µM and only minimal effects below 1 µM, demonstrating donor-independent resistance of IL-15-activated NK cells to AZD4320 (Fig. 5C).

After confirming that AZD4320 does not kill activated NK cells, we analyzed dose-response matrices to quantify combination effects (Fig. 5D, Supplementary Fig. 12A, B). In all T-ALL cell lines, the combination was more effective than single treatments, with enhanced cell death rates in all lines, except for the BE-13 cell line, which is resistant to both AZD4320 and NK cells.

**Fig. 6:**
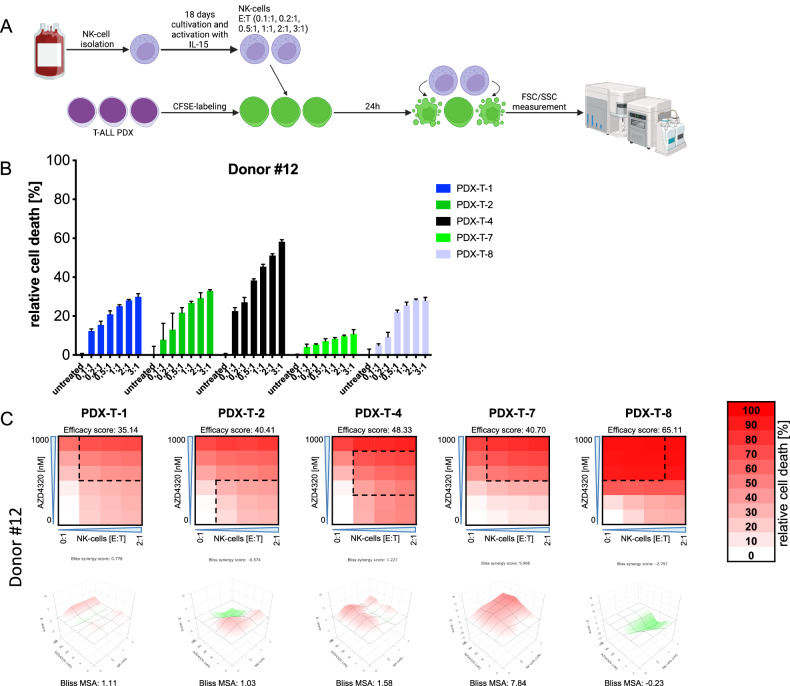
Sensitivity of T-ALL PDX Samples to NK cell-mediated killing and combinations of NK cells with AZD4320. **A** Graphical scheme of NK cell cytotoxicity assays for PDX samples. NK cells were isolated from buffy coats derived from healthy donors and amplified and activated for 18 days in medium containing IL-15 in vitro. Before co-incubation, T-ALL PDX samples were stained with CFSE to differentiate NK cells from leukemia cells using flow cytometry. After staining, T-ALL samples were incubated at increasing effector-to-target (E:T)-ratios (0.1:1, 0.2:1, 0.5:1, 1:1, 2:1 and 3:1) for 24 h before analysis of cell death by flow cytometry using FSC/SSC criteria. Created with BioRender.com. **B** Relative cell death rates of T-ALL PDX samples are shown after incubation with NK cells for 24 h. Experiments were performed in triplicate using NK cells from *N* = 2 different donors, results from one representative donor (#12) are shown. Data points show mean values and error bars standard deviations. **C** T-ALL PDX samples were exposed for 24 h to increasing concentrations of AZD4320 (5, 50, 250, 500, 1000 nM) and/or increasing E:T-ratios of NK cells (0.5:1, 1:1, 2:1) before analysis of cell death by flow cytometry using FSC/SSC criteria. Heatmaps (upper panels) show relative cell death rates for one representative experiment in triplicates of *N* = 2 experiments with NK cells from different donors. Efficacy scores were calculated as means of all normalized cell death rates across the matrix. Interaction landscapes of the combination effects are shown in the respective lower panels. Bliss synergy scores and most synergistic area (MSA) scores were calculated using SynergyFinder. Bliss synergy scores of less than −10 indicate antagonism, scores between −10 and 10 indicate additive effects and scores above 10 indicate synergism.

To confirm these data, we additionally evaluated the effectiveness of NK cells and their combination with AZD4320 in five T-ALL PDX samples. The PDX samples also displayed heterogeneous sensitivity to NK cell-mediated killing, with consistent response patterns observed across two independent NK cell donors (Fig. 6A, B, Supplementary Fig. 13A). Combination experiments of NK cells with AZD4320 yielded results similar to those observed in cell lines, with additive effects in all PDX samples, independent of the donor used (Fig. 6C, Supplementary Fig. 13B). Although PDX samples required higher drug concentrations and E:T-ratios to achieve levels of cell death comparable to cell lines, this may reflect the fact that all PDX samples were derived from relapsed or ultimately relapsing patients, mostly being characterized by drug resistance (Supplementary Fig. 7).

While no Bliss score above 10 (indicating synergy) was observed, additive effects were consistently detected across all cell lines and PDX samples and across donors, supporting the combination of NK cells and BCL-XL inhibition as a potential therapeutic strategy.

## Discussion

Dysregulation of the mitochondrial apoptosis pathway is found in many cancers, including T-ALL [14, 15]. Targeting this pathway with BH3-mimetics has shown promising preclinical and clinical results. The selective BCL-2 inhibitor venetoclax is approved for CLL and AML [20, 53] and is being investigated in other cancers including ALL [54–56]. However, not all cancers are sensitive to BCL-2 inhibition, and the emergence of resistance has been reported [57–59]. Thus, BH3-mimetics targeting other anti-apoptotic BCL-2 family members, such as MCL-1 inhibitors, BCL-XL inhibitors and dual BCL-2/BCL-XL inhibitors, are being explored in preclinical and clinical research [18, 29, 60].

In this study, we first examined the sensitivity of T-ALL cell lines to different BH3-mimetics. While most cell lines were insensitive to venetoclax (BCL-2) and AZD5991 (MCL-1), sensitivity to A1331852 (BCL-XL) and AZD4320 (BCL-2/BCL-XL) was heterogeneous. Notably, the ETP-ALL cell line Loucy showed a distinct profile with sensitivity to venetoclax but resistance to A1331852, consistent with previous reports indicating higher BCL-2 dependence in ETP-ALL compared to non-ETP-ALL [24, 47]. Surprisingly, we found that A1331852 was more effective than AZD4320 in all T-ALL cell lines except Loucy, despite AZD4320 additionally targeting BCL-2. This is in contrast to our findings in BCP-ALL, where AZD4320 was more effective than both venetoclax and A1331852 [61]. While BCL-XL is known to mediate resistance to BCL-2 inhibition in BCL-2-dependent cells [62, 63], our data suggest that the reverse is not the case in BCL-XL-dependent cells.

Analysis of apoptosis induction by Annexin V/PI staining and Caspase-Glo 3/7 assays confirmed induction of apoptosis in T-ALL in response to BH3-mimetics, with most cells being in late apoptosis (Annexin V/PI double-positive [48]) after 48 h. Interestingly, although caspase 3/7 activity indicated apoptosis induction, the extent of caspase activation did not correspond to the extent of cell death. This observation aligns with recent studies demonstrating that cells can survive executor caspase activation via a process called anastasis [64–66].

To identify functional parameters to predict sensitivity to BH3-mimetics in T-ALL, we performed BIM immunoprecipitation and baseline BH3-profiling. Total BCL-2 protein levels and BCL-2/MCL-1 and BCL-2/BCL-XL ratios were significantly correlated with venetoclax and AZD4320 sensitivity, but BCL-XL and MCL-1 levels did not predict sensitivity to their respective inhibitors. Instead, A1331852 sensitivity was only significantly correlated with BCL-XL-BIM protein complexes, suggesting that while BCL-2 levels predict BCL-2 inhibitor sensitivity [49, 67, 68], this is not clearly the case for the other anti-apoptotic proteins. Similar findings have been reported in neuroblastoma where BCL-2 expression was associated with venetoclax sensitivity, while BCL-XL and MCL-1 did not predict sensitivity to their inhibitors [69]. Additionally, also other pro-apoptotic BCL-2 family members besides BIM may interact with anti-apoptotic BCL-2 family proteins and influence sensitivity. Overall, these data suggest that sensitivity to BH3-mimetics is not solely determined by target protein levels, but rather by the broader interaction network between pro- and anti-apoptotic proteins. BH3-profiling better reflects this by measuring cytochrome c release downstream of the BCL-2 family proteins [70, 71]. Accordingly, baseline BH3-profiling showed BCL-2 dependency only in Loucy and BCL-XL dependency in the other cell lines, while low MS1 priming indicated insensitivity to MCL-1 inhibition in all cell lines. Conclusively, our findings suggest BH3-profiling as a potential marker of response for BH3-mimetics, while target protein levels largely reflect BCL-2 dependency but not dependency on BCL-XL or MCL-1.

Shifts in apoptotic dependency, such as upregulation of other anti-apoptotic proteins, have been described as causes of BH3-mimetic resistance [58, 62, 63, 72]. To determine whether such shifts are also found in T-ALL, we used dynamic BH3-profiling. Our analysis revealed an increased MCL-1 dependency after AZD4320 pre-incubation, whereas AZD5991 pre-incubation increased BCL-2 dependency exclusively in Loucy and BCL-XL dependency in the other cell lines. We and others have previously observed in other malignancies that such shifts in apoptotic dependence can predict effective combination effects [61, 73]. Therefore, we next investigated the molecular effects of the combination of AZD4320 and AZD5991 by western blot and BIM immunoprecipitation. Notably, the compensatory binding of MCL-1 to BIM under AZD4320 single treatment was prevented by co-treatment with AZD5991. In addition, the combination reduced BIM-bound BCL-2 and, most prominently, BCL-XL, supporting a synergistic interaction of AZD4320 and AZD5991. Treatment response varied across T-ALL cell lines, suggesting cell line-specific differences in dependence on specific anti-apoptotic BCL-2 family proteins. While BIM-binding patterns were largely consistent with dynamic BH3-profiling, other BH3-only proteins that interact with anti-apoptotic BCL-2 family members and additional protein loss through cell death are also likely to contribute to the effects observed here. However, BIM, which can bind all major anti-apoptotic BCL-2 family proteins and directly activate BAX and BAK, is widely regarded as a key regulator of apoptosis [74, 75].

To confirm these findings, we performed dose-response matrix analyses and calculated Bliss synergy scores, which confirmed synergism between AZD4320 and AZD5991 in all cell lines, with effectiveness at lower concentrations than the drugs alone. Comparing this combination to venetoclax + AZD5991 and A1331852 + AZD5991, we found that venetoclax was ineffective in all cell lines except Loucy and the high BCL-2-expressing ALL-SIL, though only at high concentrations. This is in contrast to BCP-ALL and AML, where venetoclax + MCL-1 inhibition can restore sensitivity [63, 76], suggesting that T-ALL cells are primarily less dependent on BCL-2. This is further supported by our observation that the combination of venetoclax and AZD5991 does not disrupt BCL-XL–BIM binding in typical T-ALL cell lines. In contrast, the ETP-ALL cell line Loucy, which is BCL-2-dependent and sensitive to the combination, exhibits very low levels of BIM-bound BCL-XL already at baseline. The A1331852 + AZD5991 combination was modestly more effective than AZD4320 + AZD5991 in all cell lines except Loucy, aligning with our EC_50_ measurements, were A1331852 outperformed AZD4320 in BCL-XL-dependent cell lines. This is likely due to higher BCL-XL binding affinity of A1331852 than AZD4320 [30, 34]. However, BCL-XL-selective inhibitors like A1331852 are known to cause severe thrombocytopenia [30, 31], limiting clinical use. In contrast, AZD4320 and its drug-dendrimer conjugate AZD0466 allow for weekly dosing with only transient thrombocytopenia that recovers within less than a week [34, 77]. Based on these factors, we decided to prioritize the AZD4320 combination for further analysis.

Since PDX models derived from patient leukemias better reflect patient characteristics and disease features than cell lines [78, 79], we used PDX samples to validate our findings. Similar to cell lines, most T-ALL PDX samples were resistant to venetoclax, except for one PDX sample derived from an ETP-ALL patient, confirming the general resistance of T-ALL to BCL-2 inhibition. For A1331852 and AZD4320, we observed heterogeneous sensitivity with no significant difference in their efficacy. Interestingly, some PDX samples were sensitive to AZD5991 in contrast to cell lines, indicating that T-ALL can be responsive to MCL-1 inhibition. Importantly, some PDX samples were resistant to all tested BH3-mimetics alone, leading us to assess the effects of combined inhibition using AZD4320 and AZD5991. Dose-response matrix analyses revealed synergy in all five PDX samples tested, potentially enabling the use of lower concentrations of the individual drugs to achieve anti-leukemia activity. By reducing individual drug concentrations, combination therapy may lower potential side effects [80–82].

Since immunotherapy plays an emerging role in the treatment of leukemia patients, especially in the relapsed or refractory setting, we aimed to investigate the interaction of BH3-mimetics with cellular therapies in T-ALL. As the use of CAR-T cells in T-ALL remains challenging due to shared antigens between CAR-T and malignant T-ALL cells, leading to CAR-T cell fratricide, NK cell-based immunotherapy is a promising alternative [83]. Interestingly, synergistic activity between BH3-mimetics and NK cell-mediated killing has been reported in other cancers, namely in AML and rhabdomyosarcoma [41, 42]. Analyzing the sensitivity of T-ALL to NK cell-mediated cytotoxicity, we found sensitivity in the majority of cell lines and PDX samples tested with stable NK cell donor-independent sensitivity patterns, consistent with previous findings [84]. Notably, we found an association between the gene expression levels of regulators of NK cell-mediated killing in T-ALL cell lines and their sensitivity to NK cells. We found that exposure of leukemia cells to NK cells caused changes in apoptotic dependencies, providing a rationale for combining NK cells with BH3-mimetics. Importantly, IL-15-activated NK cells were not sensitive to AZD4320, in line with previous reports showing insensitivity of activated NK cells towards other BH3-mimetics [41, 42]. Finally, when combining NK cells with AZD4320 in T-ALL cell lines and PDX samples, using dose-response matrix analyses, we identified additive effects, suggesting that this combination is suitable for further evaluation, particularly considering the good safety profile of NK cells [85].

Taken together, we found insensitivity of T-ALL to inhibitors of MCL-1 (AZD5991) and BCL-2 (venetoclax), except for ETP-ALL, which was sensitive to venetoclax. Sensitivity to BCL-XL (A1331852) and BCL-2/BCL-XL (AZD4320) inhibition was heterogeneous and could be predicted by BH3-profiling. Furthermore, dynamic BH3-profiling indicated synergism when combining different BH3-mimetics. Finally, we found heterogeneous sensitivity of T-ALL to NK cell-mediated killing, which could be further enhanced by BH3-mimetics, thereby providing evidence for further investigation.

## Supplementary information

Supplemental material

Original Data

## Data Availability

The gene expression sequencing data have been deposited in the NCBI Sequence Read Archive (SRA) under BioProject accession number PRJNA1263374. The data are available at https://dataview.ncbi.nlm.nih.gov/object/PRJNA1263374. All other data are available from the corresponding author upon reasonable request.
