# Supplementary material for: Therapeutic potential of BH3-mimetics and NK cell-mediated immunotherapy in T-ALL
Source: Cell Death Dis. 2026 Apr 4;17(1):387. doi: 10.1038/s41419-026-08698-x (PMC13068887; doi:10.1038/s41419-026-08698-x)

Supplementary information to

Therapeutic potential of BH3 mimetics and NK cell-mediated immunotherapy in T-ALL

Characterization of patient-derived xenograft samples

Genomic DNA of T-ALL PDX cells was prepared with the QIAamp DNA Blood Mini Kit (Qiagen) according to manufacturer`s instructions. The copy number variations of genes were determined by Multiplex Ligation-dependent Probe Amplification (MLPA) according to manufacturer`s instructions using the SALSA MLPA Probemix P283 T-ALL (MRC Holland, REF: P383-050R).

Cell viability assays

To determine half maximal effective concentrations (EC_{50}) values in cell lines, cells were exposed to eleven increasing concentrations (0.1, 1, 5, 10, 50, 100, 250, 500, 1000, 5000, 10000 nM) of venetoclax, A1331852, AZD4320, AZD5991 and S63845 and cell death rates were quantified by measuring propidium iodide (PI) positivity by flow cytometry after drug exposure for 48 hours. To determine the EC_{50} values in PDX samples, samples were exposed to eight increasing concentrations (1, 10, 50,100, 500, 1000, 5000, 10000 nM) of venetoclax, A1331852, AZD4320, AZD5991 and S63845 followed by quantification of cell death rates by measuring PI positivity by flow cytometry after drug exposure for 24 hours.

To assess the sensitivity of IL-15 activated NK cells to AZD4320, cells were exposed on day 18 after isolation to nine increasing concentrations of AZD4320 (1, 10, 100, 250, 500, 1000, 2500, 5000, 10000 nM). Cell death rates were quantified by measuring PI positivity by flow cytometry after drug exposure for 24 hours.

Combination effects were analyzed for both cell lines and PDX samples using dose-response matrix analyses upon exposure to seven increasing concentrations (2.5, 5, 25, 50, 250, 500, 2500 nM) of both drugs. Cell death rates were determined according to PI positivity after drug exposure for 48 hours in cell lines and based on forward/side scatter criteria after drug exposure for 24 hours in PDX samples.

To assess apoptosis induction, Annexin V/PI staining was performed. Cells were exposed to increasing drug concentrations (0.1, 5, 10, 50, 100, 250, 500, 1000, 5000 nM) for 48 hours and subsequently stained with Annexin V and PI, followed by quantification by flow cytometry.

Caspase-Glo 3/7 assays

For the analysis of caspase activation, 50 µl of the same cell suspension used for Annexin V/PI staining were taken and subjected to caspase-3/7 assays using the Caspase-Glo® 3/7 Assay System (Promega, G8090) according to manufacturer's instructions.

BH3-profiling

Baseline BH3-Profiling was performed as previously described (1, 2). Cells were stained with Zombie Violet (1:1000; 423113, Biolegend) for 15 minutes, permeabilized with digitonin and exposed to BH3-peptides for 30 minutes. Thereafter, cells were fixed (4% formaldehyde) and neutralized in N2 buffer for 10 minutes each. Finally, cells were incubated overnight with Alexa Flour 488 anti-Cytochrome c antibody (612308, Biolegend), 1:400 in intracellular staining buffer.

For dynamic BH3-Profiling, cells were exposed to AZD4320, AZD5991 or DMSO controls or for 4 hours (Loucy) or 2 hours (all others) prior to Zombie Violet staining. Drug concentrations of AZD4320 and AZD5991 were selected based on their

sensitivity in each cell line (AZD4320: Loucy (3 nM), ALL-SIL (4 nM), MOLT-4 (15 nM), BE-13 (10 nM), CCRF-CEM (50 nM), Jurkat (50 nM) / AZD5991: Loucy (250 nM), ALL-SIL (250 nM), MOLT-4 (400 nM), BE-13 (150 nM), CCRF-CEM (400 nM), Jurkat (150 nM).

BH3 profiling results were analyzed by flow cytometry on an Attune NxT flow cytometer (Thermo Fisher) using an autosampler and analyzed with FlowJo software. BH3-peptides with the following sequences were used: mBAD: Ac-LWAAQRYGRELRRMSDEFEGSFKGL-NH_2, w-HRK: Ac-WSSAAQLTAARLKALGDELHQ-NH_2, MS1: Ac-RPEIWMTQGLRRLGDEINAYYAR-NH_2.

Immunoprecipitation and immunoblotting

Western blot and immunoprecipitation were performed as previously described (3). Protein extraction was performed using lysis buffer containing 30 mM Tris-HCL, 10% glycerol and 1% Triton X-100. For immunoprecipitation, cell lysates were co-incubated with BIM (C34C5) Rabbit mAb (2933, Cell Signaling) on a rotary overnight at 4°C. On the next day, lysates were co-incubated with Protein A Agarose Beads (9863, Cell Signaling) for 2-4 hours on a rotary at 4°C. Thereafter, beads were washed with lysis buffer five times and the precipitates were used for western blot analyses using Bolt™ 12 %, Bis-Tris, 1,0 mm, Mini-Protein-Gel (Invitrogen). Primary antibodies; BCL-2 (124 Mouse mAb (15071, Cell Signaling), BCL-XL (54H6) Rabbit mAb (2764, Cell Signaling), MCL-1 (D2W9E) Rabbit mAb (94296, Cell Signaling), Bim (C34C5) Rabbit mAb (2933, Cell Signaling), α/β-Tubulin Antibody (2148, Cell Signaling), GAPDH monoclonal antibody (1D4) (ADI-CSA-335-E, Enzo) were incubated at 4°C overnight followed by secondary antibodies m-IgGk BP-HRP (sc-516102, Santa Cruz), mouse anti-rabbit IgG-HRP (sc-2357, Santa Cruz), StarBright Blue 700 Goat Anti-Rabbit IgG

(12004162, Bio-Rad), StarBright Blue 700 Goat Anti-Mouse IgG (12004159, Bio-Rad) for 1 hour at room temperature and hFAB™ Rhodamine Anti-GAPDH Primary Antibody (12004168, BIO-RAD) was applied for 1 hour at room temperature. Antibodies were generally applied in the following order BCL-2, BCL-XL, MCL-1, BIM, Tubulin or GAPDH. Immunoblots were developed by chemiluminescence and ImageJ software was used for densitometric quantification.

NK cell isolation and expansion

NK cells were isolated from Buffy coats obtained from healthy donors provided by the Institute of Transfusion Medicine at Ulm University Medical Center. First, peripheral blood mononuclear cells (PBMCs) were isolated by density gradient centrifugation. Thereafter, NK cells were enriched by negative selection using EasySep Human NK Cell Enrichment Kit (19055, STEMCELL Technologies) followed by expansion and activation with interleukin (IL)-15 (200-15, PeproTech). NK cells were cultured in NK-MACS medium (130-114-429, Miltenyi Biotec) supplemented with NK MACS Supplement (130-114-429, Miltenyi Biotec), Penicillin/Streptomycin, human plasma (provided by the Institute of Transfusion Medicine at Ulm University Medical Center) and recombinant human IL-15 (1 ng/µl stock, 1:100). Medium and IL-15 were replaced on days 4, 7, 11, 14 and 18. The purity of isolated NK cells was assessed on day 0 and on day 14 after isolation using an immunofluorescence panel (Supplementary Figure 4A, B). NK cells were identified as CD45+, CD56+ and CD3- cells. The following antibodies were used: FITC Mouse Anti-Human CD45 (555482, BD Biosciences), Pacific Blue Mouse Anti-Human CD3 (558117, BD Biosciences), CD56(My31) PE (345810, BD), CD45(2D1) AmCyan (339192, BD Biosciences), APC anti-human CD16 clone: B73.1 (360705, BioLegend) and 7-AAD (51-68981E (559925), BD Biosciences). Cell populations were evaluated using an LSR-II flow cytometer (BD Biosciences) or

an Attune NxT flow cytometer (Thermo Fisher). NK cells were used for co-culture experiments after expansion and activation with IL-15 for 18 or 21 days.

NK cell cytotoxicity assays

To measure cell death induction in ALL samples following co-culture with NK cells, ALL cells were labeled with CFSE (3 µM Stock 1:1666 for cell lines, 1:1000 for PDX, Invitrogen, 65-0850-84) according to the manufacturer's protocol. NK cells were co-cultured with T-ALL cell lines at increasing effector-to-target (E:T) ratios (0.2:1, 0.5:1, 1:1, 3:1) for 24 hours in the presence of IL-15. In order to address combination effects, T-ALL cell lines were exposed to increasing concentrations of AZD4320 (1, 10, 50, 100, 250 nM) and NK cells at increasing E:T ratios (0.1:1, 0.2:1, 0.5:1) for 24 hours in the presence of IL-15. T-ALL PDX samples were co-cultured with NK cells at increasing E:T ratios (0.1:1, 0.2:1, 0.5:1, 1:1, 2:1, 3:1) for 24 hours in the presence of IL-15. In order to address combination effects, PDX samples were exposed to increasing concentrations of AZD4320 (5, 50, 250, 500, 1000 nM) and NK cells at increasing E:T ratios (0.5:1, 1:1, 2:1) for 24 hours in the presence of IL-15. Cell death rates were determined by flow cytometry analysis by assessing FSC/SSC criteria in CFSE-labeled ALL cells **(**Supplementary Figure 10C**)**.

RNA-sequencing of T-ALL cell lines

T-ALL cell lines were supplied with fresh medium 6 hours prior to preparation and cultured at 37°C and 5% CO_2. Cell pellets were collected and total RNA isolation was performed using Quick-RNA MiniPrep kit (R1055, ZYMO Research). RNA-sequencing and data normalization were performed by Novogene GmbH (Munich, Germany). Sequencing was performed on a NovaSeq X Plus Series (PE150) after mRNA library

preparation using poly A enrichment. All sequencing data are available under BioProject PRJNA1263374.

Dynamic BH3-profiling after treatment of T-ALL with NK cells

To measure changes in apoptotic dependency after exposure to IL-15 activated NK cells, target cells were first stained with CFSE as described above. Afterwards, cells were co-incubated with NK cells (E:T-ratios as indicated) for 4 hours and BH3-profiling was performed as described above, using Alexa Fluor 647 anti-Cytochrome c antibody (612310, BioLegend) for cytochrome c detection.

Supplementary References:

1. Montero J, Sarosiek KA, DeAngelo JD, Maertens O, Ryan J, Ercan D, et al. Drug-induced death signaling strategy rapidly predicts cancer response to chemotherapy. Cell. 2015;160(5):977-89.

2. Ryan J, Montero J, Rocco J, Letai A. iBH3: simple, fixable BH3 profiling to determine apoptotic priming in primary tissue by flow cytometry. Biol Chem. 2016;397(7):671-8.

3. Seyfried F, Stirnweiß FU, Niedermayer A, Enzenmüller S, Hörl RL, Münch V, et al. Synergistic activity of combined inhibition of anti-apoptotic molecules in B-cell precursor ALL. Leukemia. 2022;36(4):901-12.

Supplementary Figure 1: Gating strategy and representative FACS blots for dose response measurements in T-ALL cell lines

T-ALL cell lines were exposed for 48 hours to increasing concentrations (0.1, 1, 5, 10, 50, 100, 250, 500, 1000, 5000, 10000 nM) of venetoclax, A1331852, AZD4320 or AZD5991 before analysis of cell death by propidium iodide staining and flow cytometry. (A) Gating strategy for BH3-mimetic dose response measurements in T-ALL cell lines. CCRF-CEM DMSO control is shown as a representative. (B) FACS blots of BH3-mimetic dose response measurements for all drugs. DMSO control (same as in (A)) of venetoclax measurement and 1000 nM measurements of all drugs for CCRF-CEM are shown as representatives. (C) EC_{50} values corresponding to the dose response curves in Figure 1A-D, as shown in Figure 1E-G.

A

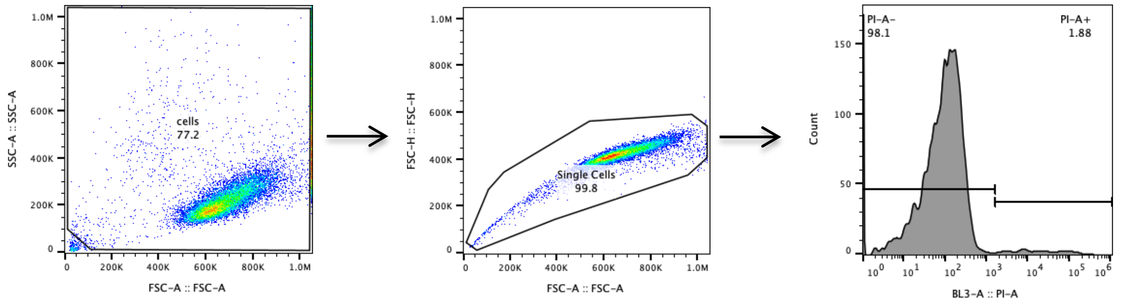

B

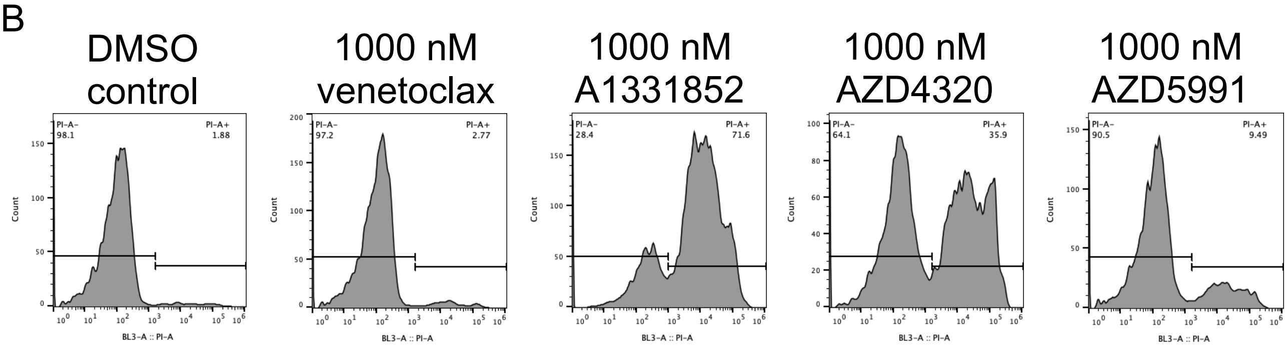

C

		Loucy	ALL-SIL	MOLT4	BE-13	CCRF-CEM	Jurkat
EC50 [nm]	**venetoclax**	64.34	2489	18203	13335	47681	19568
	**A1331852**	1036	16,86	26.36	85.01	152.3	10899
	**AZD4320**	7.633	55.04	551.1	914.1	1622	22898
	**AZD5991**	2141	3153	29034	15622	22753	1854
	**S63845**	1195	745.3	3319	4894	17087	4405

Supplementary Figure 2: Treatment with BH3 mimetics induces apoptosis markers, Annexin V and caspase 3/7

T-ALL cell lines were exposed for 48 hours to increasing concentrations (0.1, 5, 10, 50, 100, 250, 500, 1000, 5000nM) of venetoclax, A1331852, AZD4320 or AZD5991 before analysis of cell death by Annexin V and propidium iodide (PI) staining and flow cytometry as well as Caspase-Glo 3/7 assay. Three cell lines in technical triplicates. (A) Gating strategy for Annexin V PI measurements. Loucy DMSO control is shown as a representative. (B) Representative FACS blots of Annexin V PI measurements for all cell lines and drugs. (C) Dose response curves of Annexin V positive cells from the Annexin V PI staining. Curves show mean values of technical triplicates with standard deviation as error bars and connecting lines. (D) Caspase-3/7 activity resulting from Caspase-Glo 3/7 measurements. Bar graphs show mean values of technical triplicates with error bars indicating standard deviations.

A

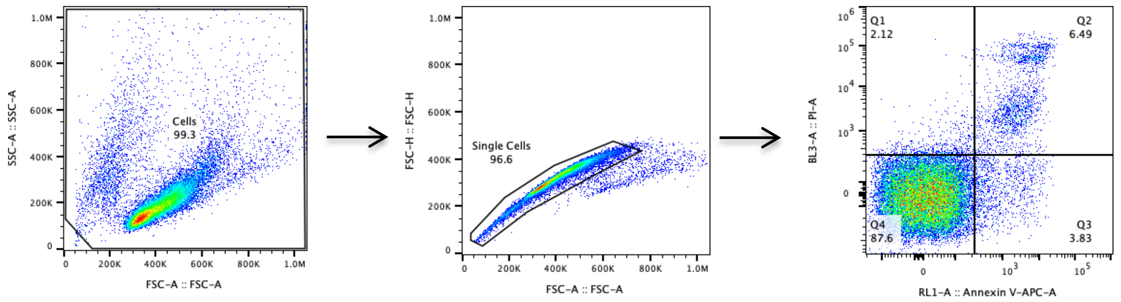

B

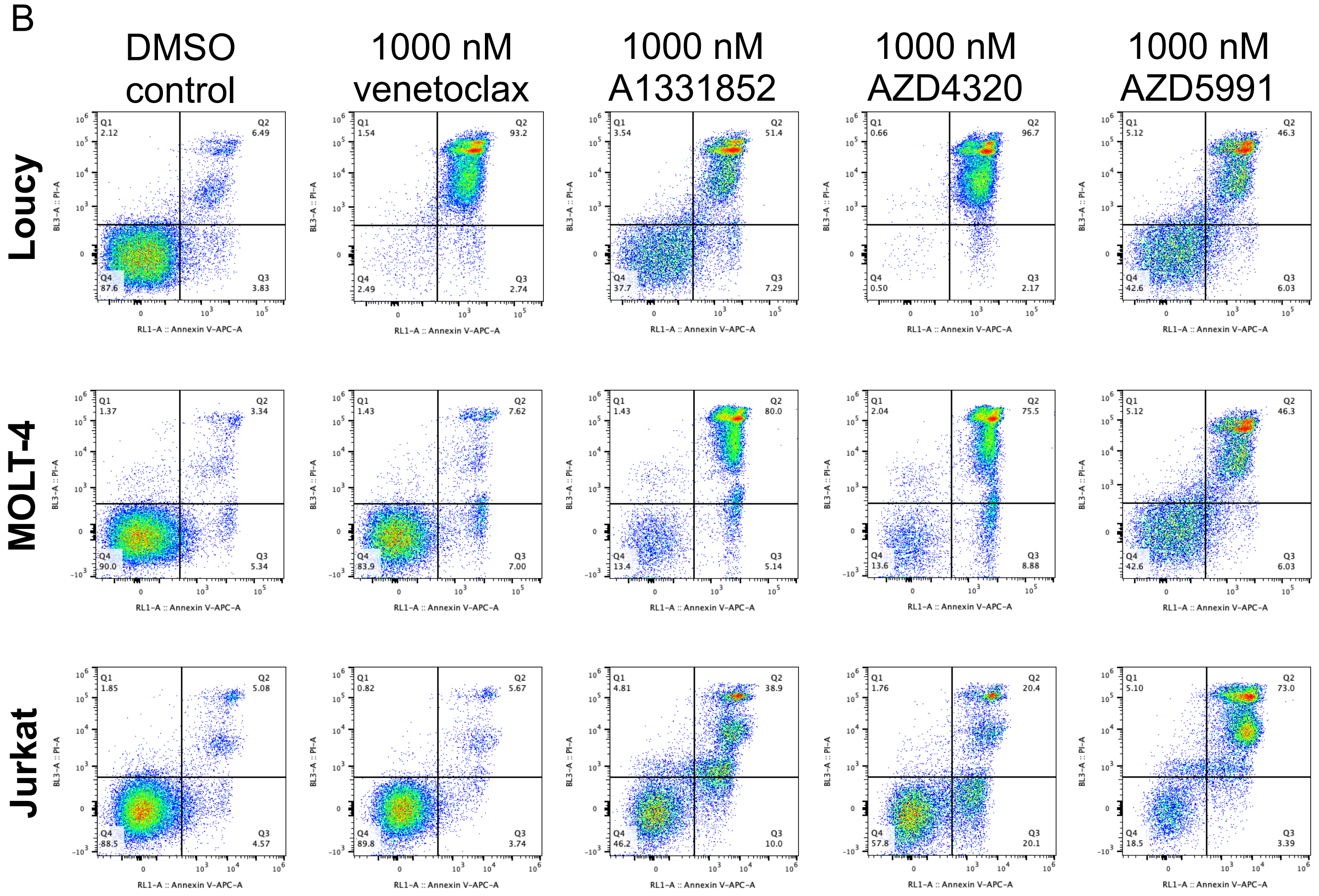

Supplementary Figure 2 continued

C

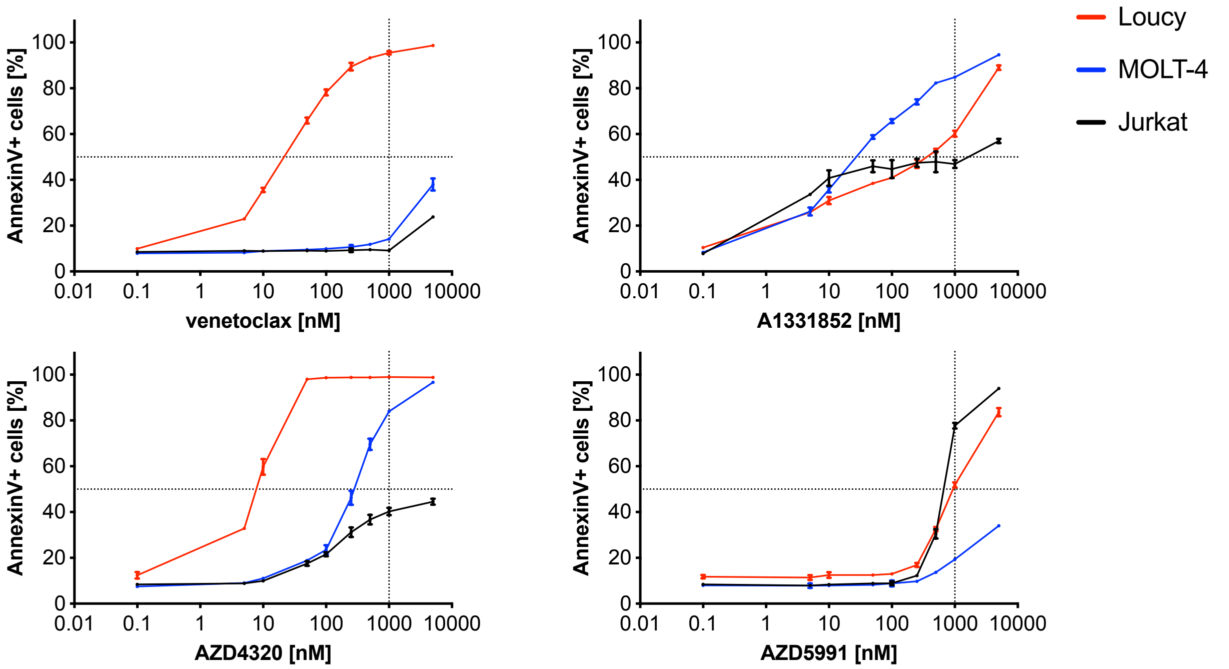

D

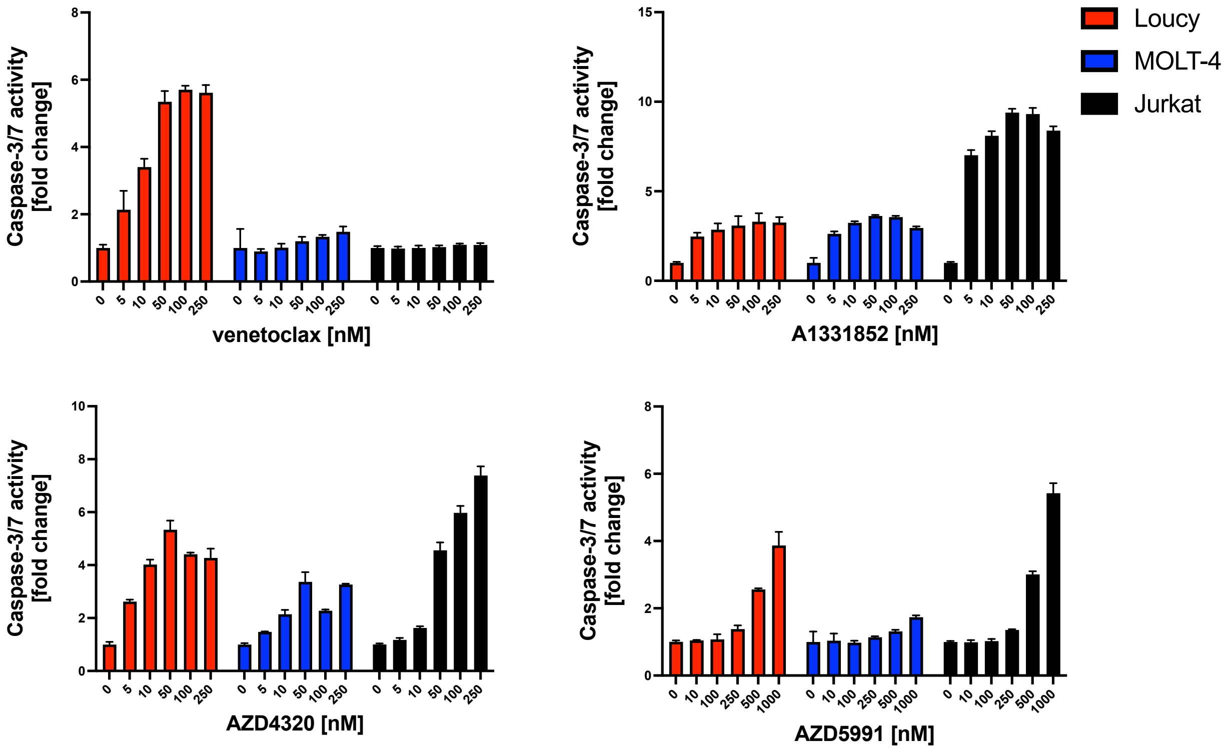

Supplementary Figure 3: Protein levels of apoptosis regulators in T-ALL

(A) Baseline protein levels and BIM protein complexes in T-ALL cell lines. Second and third Repetition of the experiment in Figure 1H are shown. Protein extracts of T-ALL cell lines were co-incubated with anti-BIM-antibody or rabbit igG for isotype controls, overnight, co-immunoprecipitation and input protein extracts were subjected to western blot. Jurkat was used for IgG isotype control in (2) and CCRF-CEM in (3). Proteins were detected using HRP labelled secondary antibodies. (B) Baseline protein levels and BIM protein complexes in T-ALL cell lines. Protein extracts of T-ALL cell lines were co-incubated with anti-BIM-antibody or rabbit igG for isotype controls overnight, co-immunoprecipitation and input protein extracts were subjected to western blot. Proteins were detected using Fluorescence labelled secondary antibodies or Rhodamine labelled GAPDH primary antibody. (C) Loucy cells were exposed for 6 hours to 20 nM AZD4320 and/or 2 µM AZD5991. BE-13 cells were exposed for 4 hours to 1 µM AZD4320 and/or 2 µM AZD5991.

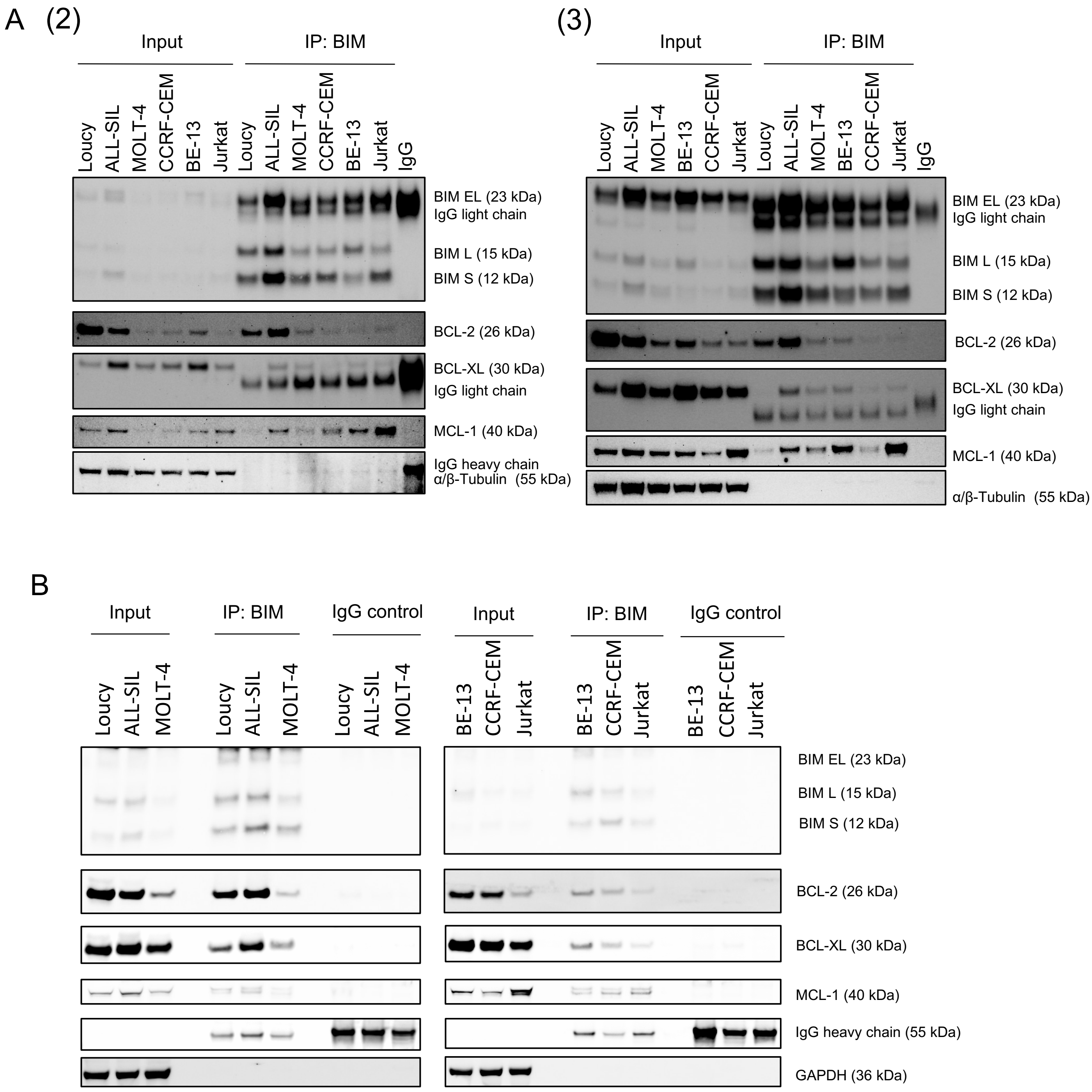

C

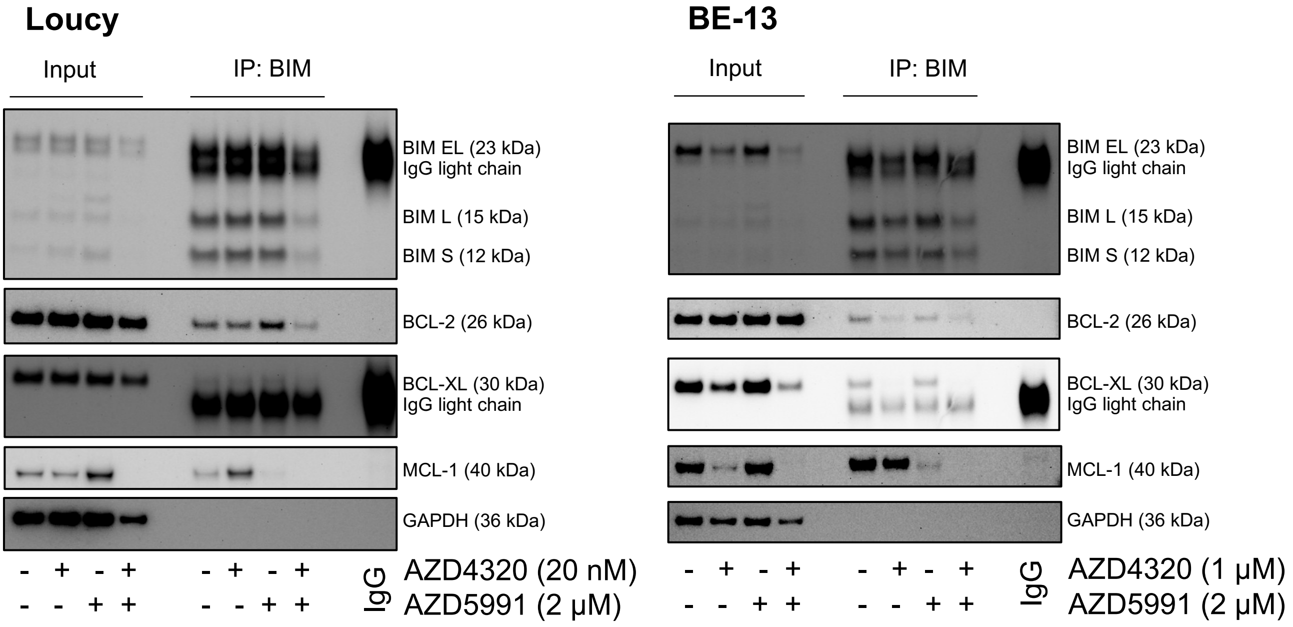
Loucy
Input
IP: BIM
BIM EL (23 kDa)
IgG light chain
BIM L (15 kDa)
BIM S (12 kDa)
BCL-2 (26 kDa)
BCL-XL (30 kDa)
IgG light chain
MCL-1 (40 kDa)
GAPDH (36 kDa)
- + - + - + - +
- - + + - - + +
IgG
AZD4320 (20 nM)
AZD5991 (2 μM)
BE-13
Input
IP: BIM
BIM EL (23 kDa)
IgG light chain
BIM L (15 kDa)
BIM S (12 kDa)
BCL-2 (26 kDa)
BCL-XL (30 kDa)
IgG light chain
MCL-1 (40 kDa)
GAPDH (36 kDa)
- + - + - + - +
- - + + - - + +
IgG
AZD4320 (1 μM)
AZD5991 (2 μM)

Supplementary Figure 4: Gating strategy and representative FACS blots for BH3-profiling measurements

T-ALL cell lines were incubated with BH3-peptides before fixing and cytochrome c staining. Cytochrome c release after peptide exposure was analyzed by flow cytometry and values were normalized to alamethicin as positive control and DMSO as negative control. (A) Shows the gating strategy used to determine cytochrome c release in response to BH3-peptides. DMSO control of Loucy in baseline BH3 profiling is shown as a representative. For dynamic BH3-profiling the same gating strategy was used. (B) Representative FACS blots of all peptides and controls. Loucy basline BH3 profiling is shown as a representative.

A

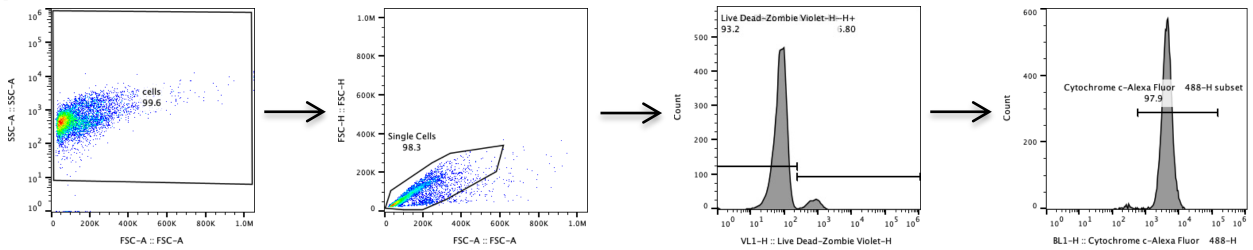

B

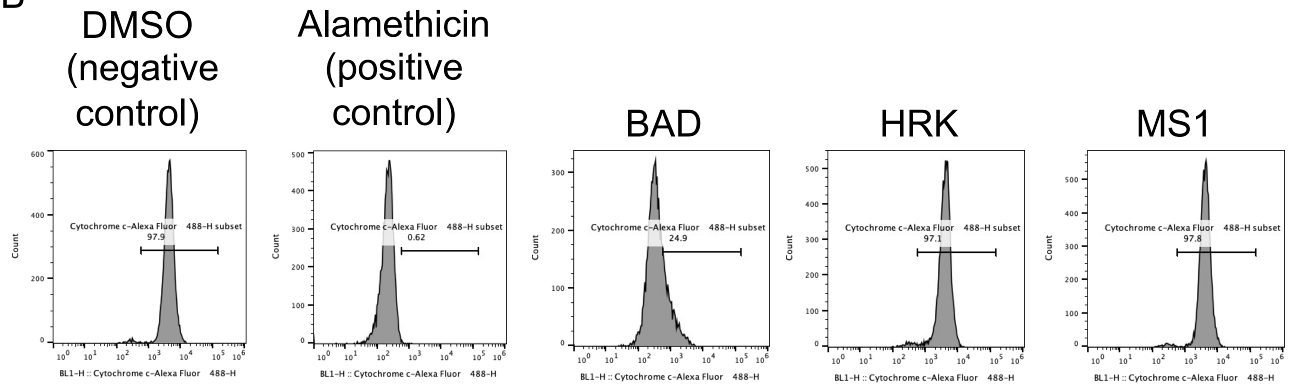

Supplementary Figure 5: Synergism of AZD4320 and AZD5991 in T-ALL cell lines
Cell death was assessed upon exposure of T-ALL cell lines to increasing concentrations (2.5, 5, 25, 50, 250, 500, 2500 nM) of venetoclax, A1331852, AZD4320 and/or AZD5991 for 48 hours. The heatmaps show dose-response matrix analyses based on cell death assessed by propidium iodide (PI) staining of the cell lines (A) ALL-SIL (B) BE-13 (C) CCRF-CEM and (D) Jurkat. Mean values of triplicates are shown in the heatmaps. The dashed lines indicate the most synergistic areas. Efficacy scores were calculated as means of all normalized cell death rates across the matrix. Interaction landscapes of the combination effects are shown in the respective lower panels. Bliss synergy scores and most synergistic area (MSA) scores were calculated using SynergyFinder. Bliss synergy scores of less than -10 indicate antagonism, scores between -10 and 10 indicate additive effects and scores above 10 indicate synergism.

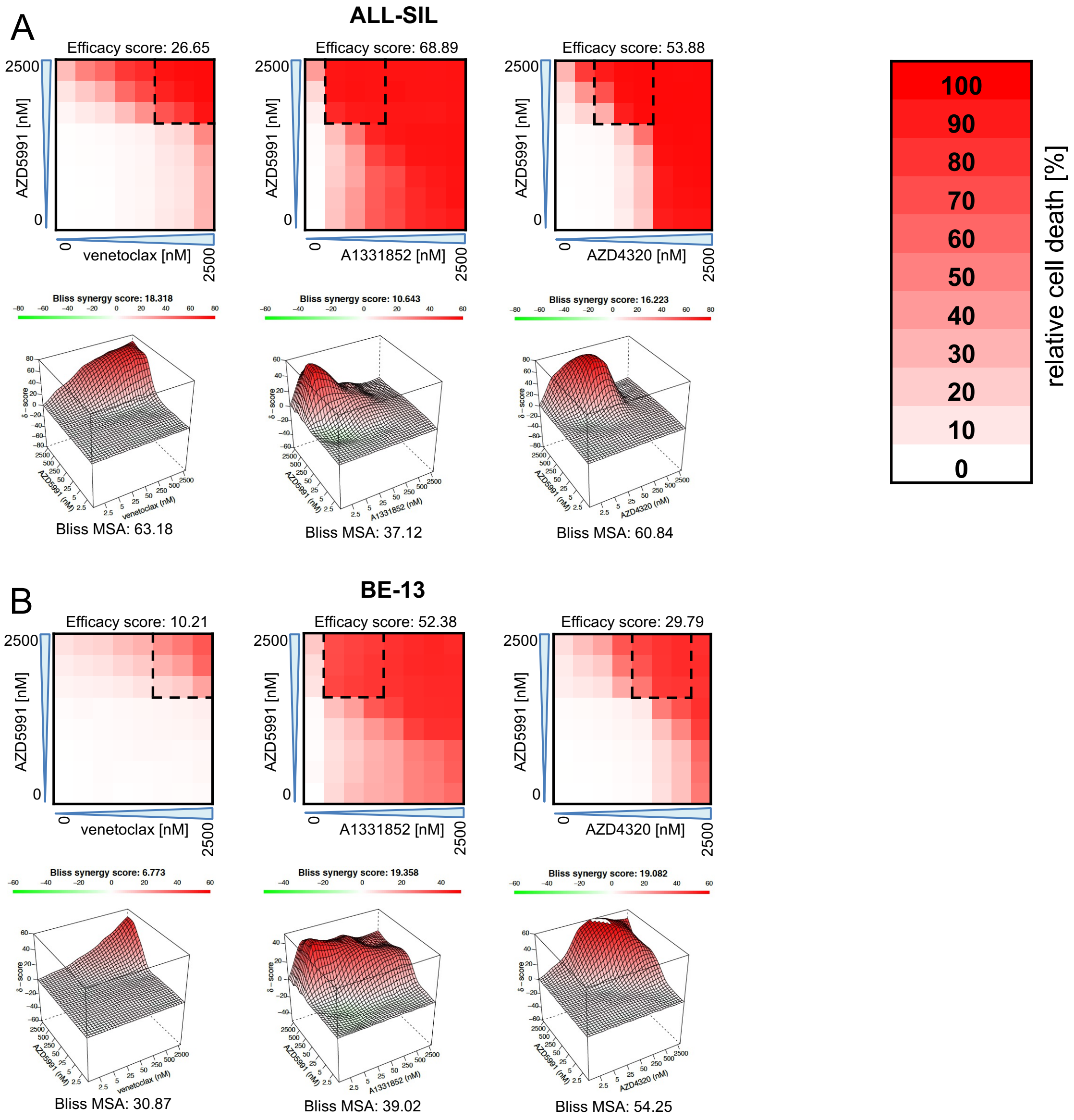

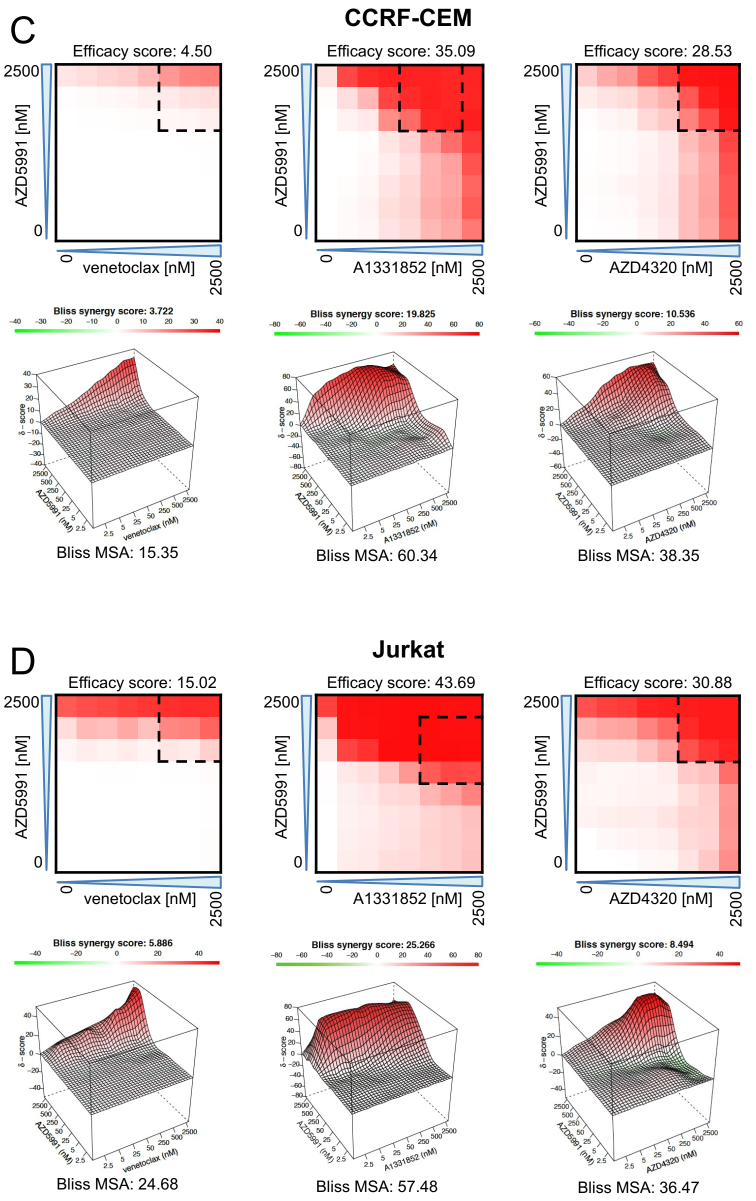
C
CCRF-CEM
Efficacy score: 4.50
Efficacy score: 35.09
Efficacy score: 28.53
AZD5991 [nM]
venetoclax [nM]
A1331852 [nM]
AZD4320 [nM]
Bliss synergy score: 3.722
Bliss synergy score: 19.825
Bliss synergy score: 10.536
Bliss MSA: 15.35
Bliss MSA: 60.34
Bliss MSA: 38.35
D
Jurkat
Efficacy score: 15.02
Efficacy score: 43.69
Efficacy score: 30.88
Bliss synergy score: 5.886
Bliss synergy score: 25.266
Bliss synergy score: 8.494
Bliss MSA: 24.68
Bliss MSA: 57.48
Bliss MSA: 36.47

Supplementary Figure 6: Protein levels of apoptosis regulators in T-ALL after venetoclax treatment

Baseline protein levels and BIM protein complexes in T-ALL cell lines. (A) Loucy cells were exposed for 6 hours to 60 nM venetoclax and/or 2 µM AZD5991. (B) ALL-SIL cells were exposed for 4 hours to 1 µM venetoclax and/or 1 µM AZD5991. (C) BE-13 cells were exposed for 4 hours to 1 µM venetoclax and/or 2 µM AZD5991. Afterwards, Protein extracts of T-ALL cell lines were co-incubated with anti-BIM-antibody overnight, co-immunoprecipitation and input protein extracts were subjected to western blot. IPs were performed once per cell line in three different cell lines. (D) Quantification of BCL-2, BCL-XL and MCL-1 protein levels from the western blots shown in A-C. Input proteins were normalized to the loading controls TUBULIN or GAPDH, while IP proteins were normalized to the sum of BIM_{EL}, BIM_{L}, and BIM_{S}. Densitometric quantification was performed using ImageJ.

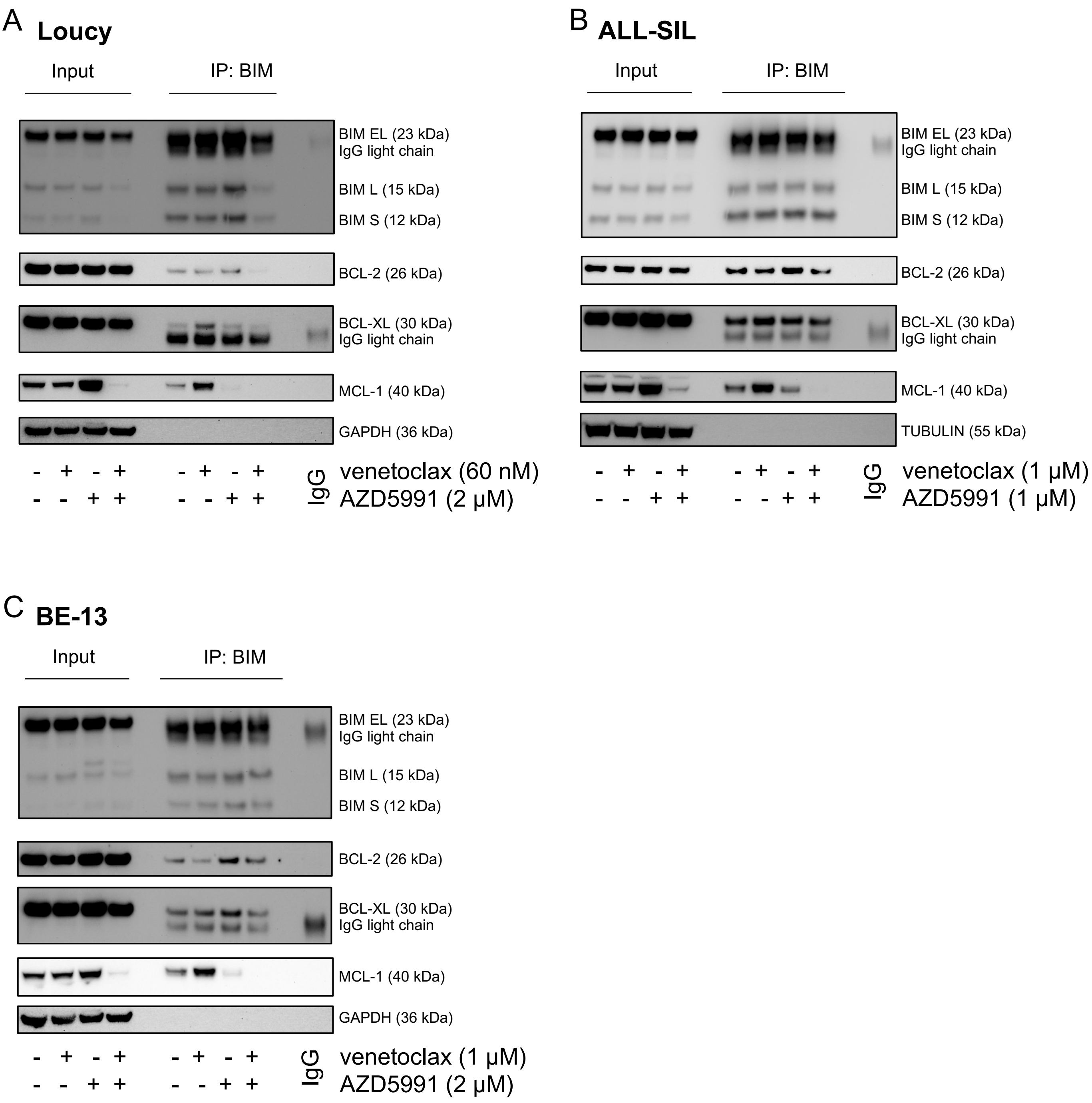

Supplementary Figure 6

D

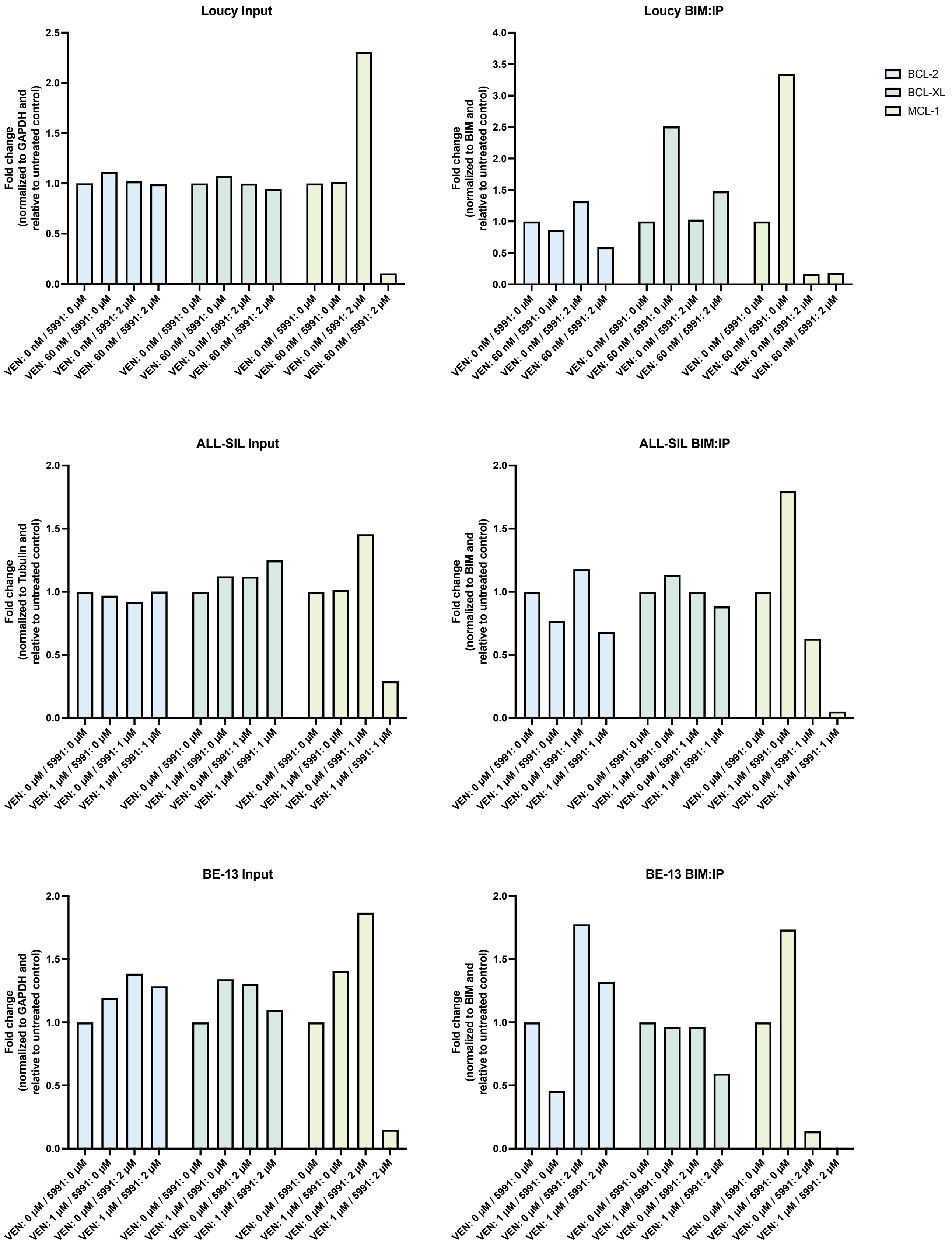

Supplementary Figure 7: Genetic alterations of T-ALL patient derived xenograft (PDX) samples

DNA isolated from the ten T-ALL PDX samples was analyzed by Multiplex Ligation-dependent Probe Amplification (MLPA) according to manufacturer`s instructions using the SALSA MLPA Probemix P283 T-ALL. Samples are ordered according to EC_{50} of AZD4320.

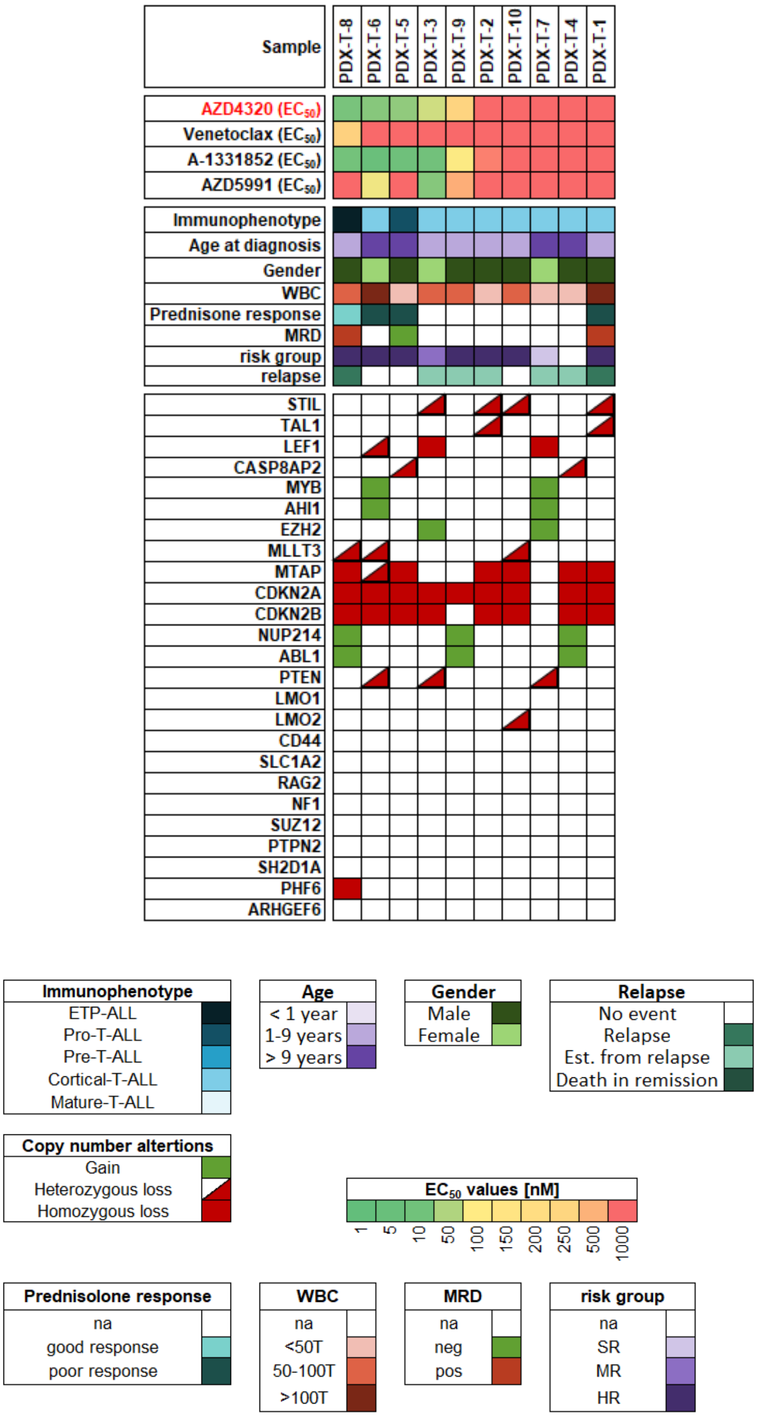

Supplementary Figure 8: Gating strategy and representative FACS blots for dose response measurements in T-ALL PDX samples

T-ALL PDX samples were exposed for 24 hours to increasing concentrations (1, 10, 50, 100, 500, 1000, 5000, 10000 nM) of venetoclax, A1331852, AZD4320 or AZD5991 before analysis of cell death by propidium iodide staining and flow cytometry. (A) Gating strategy for BH3-mimetic dose response measurements in T-ALL PDX-samples. PDX-T-4 DMSO control is shown as a representative. (B) FACS blots of BH3-mimetic dose response measurements for all drugs. DMSO control (same as in (A)) of PDX-T-4 measurement and 1000 nM measurements of all drugs for PDX-T-4 are shown as representatives. (C) EC_{50} values corresponding to the dose response curves in Figure 4A-D, as shown in Figure 4E-G.

A

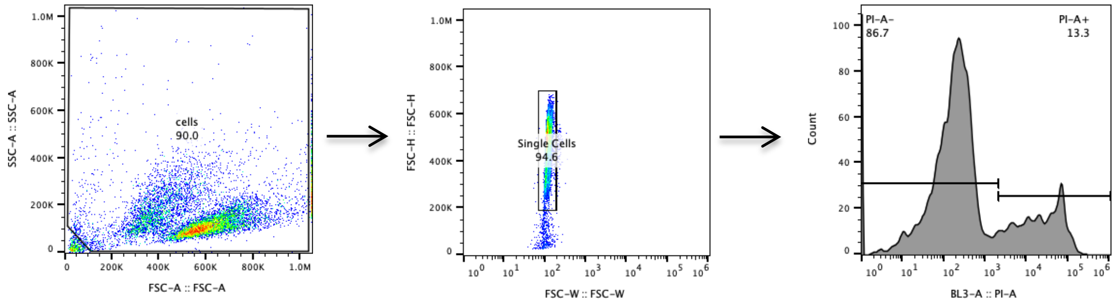

B

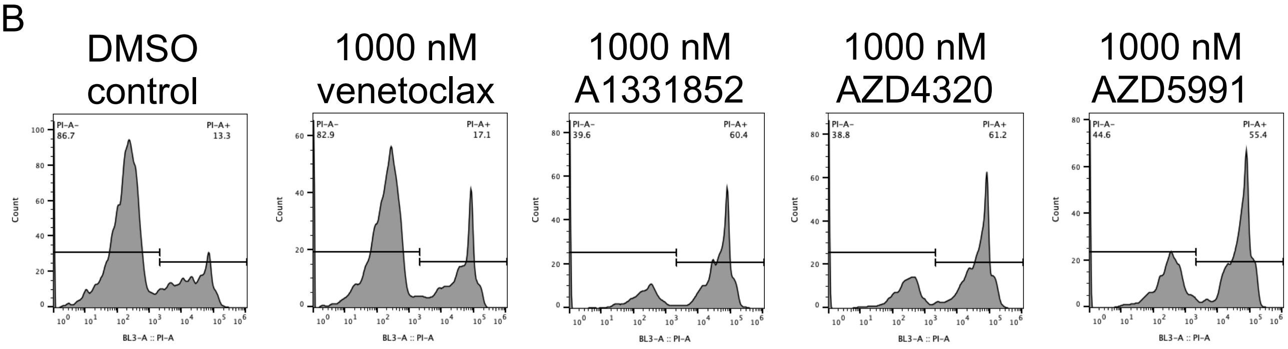

C

EC50 [nm]		PDX-T-1	PDX-T-2	PDX-T-3	PDX-T-4	PDX-T-5	PDX-T-6	PDX-T-7	PDX-T-8	PDX-T-9	PDX-T-10
	**venetoclax**	96014	79761	19932	22627	11658	4500	44578	284,2	124965	8131
	**A1331852**	2344	842,8	9,664	1001	7,572	4,504	5636	11,09	98,13	2621
	**AZD4320**	4598	1181	69,58	3050	29,57	22,89	2264	14,12	262,1	1695
	**AZD5991**	509271	84265	22,58	4154	4521	88,34	12449	14760	525,8	6030
	**S63845**	100230	19365	69,7	4725	3918	183,1	9560	456	578,8	2646

Supplementary Figure 9: Activity of S63845 in T-ALL cell lines
(A) T-ALL cell lines were exposed for 48 hours to increasing concentrations (0.1, 1, 5, 10, 50, 100, 250, 500, 1000, 5000, 10000 nM) of S63845 before analysis of cell death by propidium iodide staining and flow cytometry. N=3 independent experiments in triplicates. (B) Association of the EC_{50} values of S63845 with those of AZD5991 in all cell lines. Spearman correlation (two-tailed); r, correlation coefficient; p, significance. (C) T-ALL PDX-samples were exposed for 24 hours to increasing concentrations (1, 10, 50, 100, 500, 1000, 5000, 10000 nM) of S63845 before analysis of cell death by propidium iodide staining and flow cytometry. Experiments were performed in triplicates. (D) Association of the EC_{50} values of S63845 with those of AZD5991 in PDX-samples. Spearman correlation (two-tailed); r, correlation coefficient; p, significance. (E, F) For analysis of combination effects cell death was assessed upon exposure of T-ALL cell lines to increasing concentrations (2.5, 5, 25, 50, 250, 500, 2500 nM) of venetoclax, A1331852, AZD4320 and/or S63845 for 48 hours. The heatmaps show dose-response matrix analyses based on cell death assessed by propidium iodide (PI) staining of the cell lines (E) Loucy and (F) MOLT-4. Mean values of triplicates are shown in the heatmaps. The dashed lines indicate the most synergistic areas. Efficacy scores were calculated as means of all normalized cell death rates across the matrix. Interaction landscapes of the combination effects are shown in the respective lower panels. Bliss synergy scores and most synergistic area (MSA) scores were calculated using SynergyFinder. Bliss synergy scores of less than -10 indicate antagonism, scores between -10 and 10 indicate additive effects and scores above 10 indicate synergism.

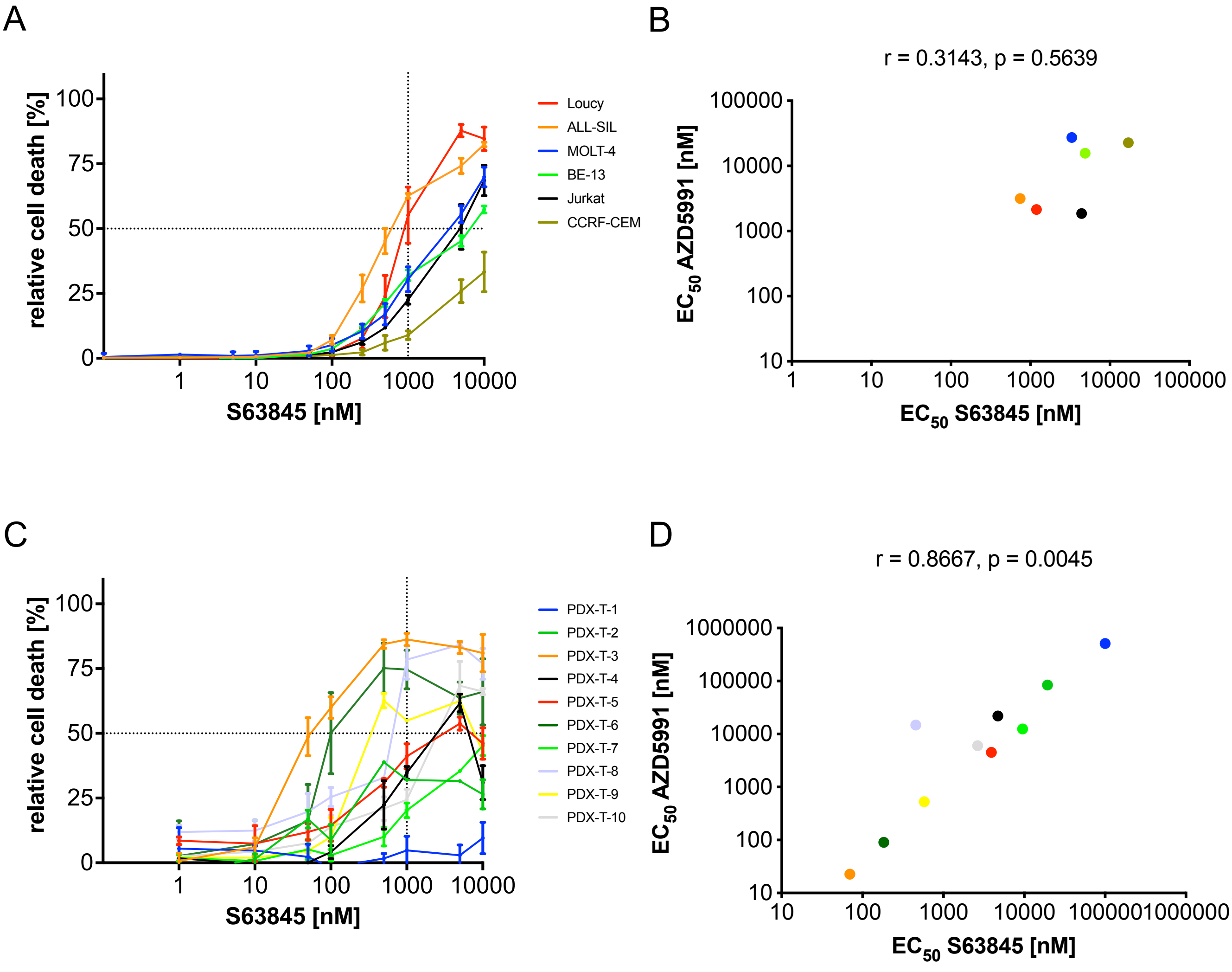

Supplementary Figure 9

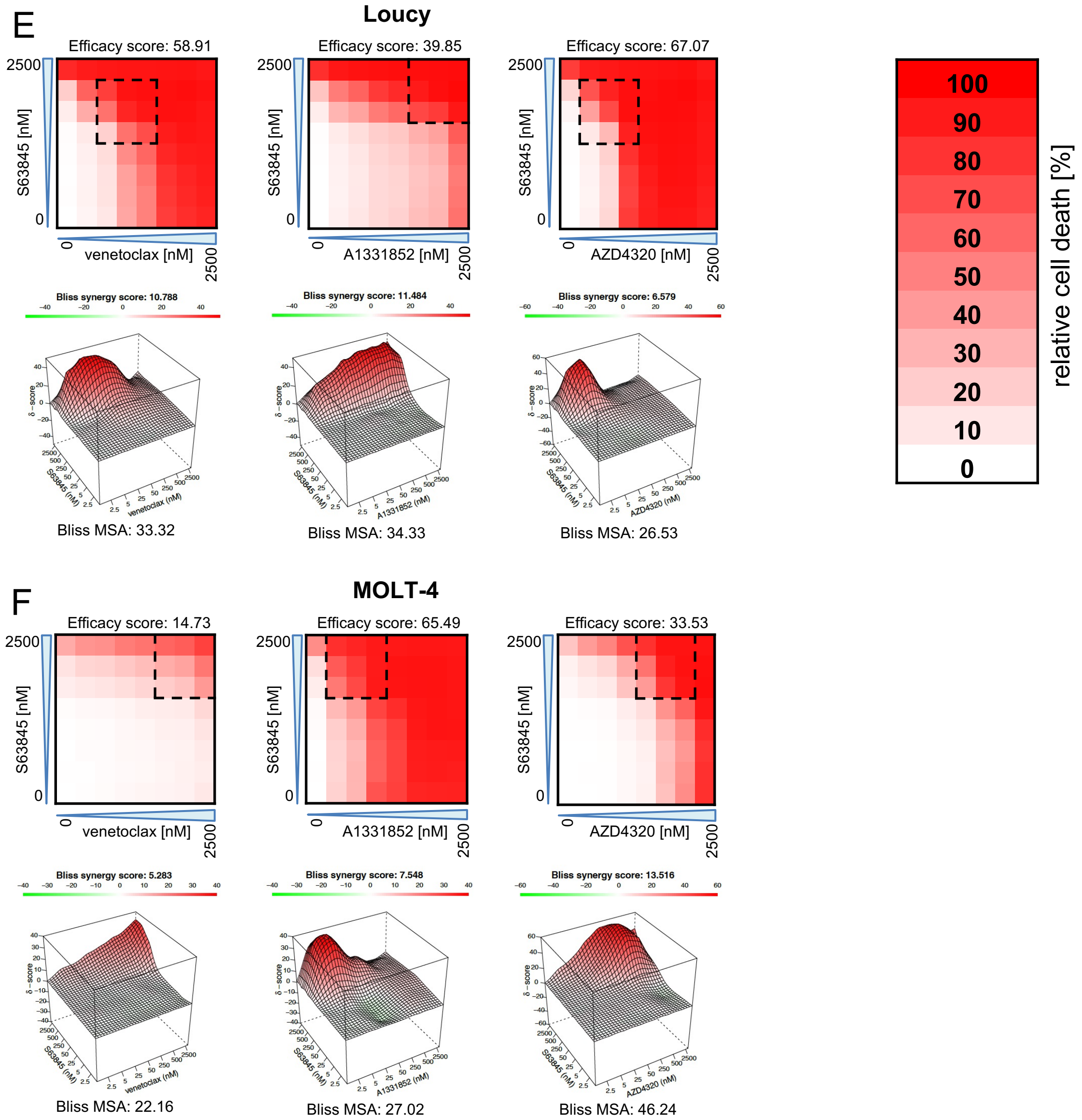

Supplementary Figure 10: Gating strategy for the assessment of NK cell purity and cell death induction in NK cell cytotoxicity assays

(A) Gating strategy for the assessment of the purity of NK cells isolated from healthy donor buffy coats. After gating for single cells, 7AAD staining is used to gate for living cells. Afterwards, the lymphocyte population is selected and gated for CD45-positive cells. The CD45-positive population is gated for CD56 and CD3, with CD56+CD3- cells representing NK cells, CD56-CD3+ cells representing T-cells and double-positive cells representing NKT-cells. CD16-positivity was assessed as an additional control. Purity check of donor #9 on day 0 is shown as a representative. (B) Purity checks on day 0 and on day 14 and donor characteristics of all donors used in this study are shown. (C) Gating strategy for the measurement of cell death induction by DAPI staining in NK cell cytotoxicity assays. First, cell debris is excluded. CFSE-staining is used to identify the target cells and to exclude the NK cells. Target cells are gated for single cells and then cell death rates are assessed by gating on DAPI-positive cells. ALL-SIL cells co-incubated with donor #1 NK cells at E:T 0.5:1 is shown as a representative.

A

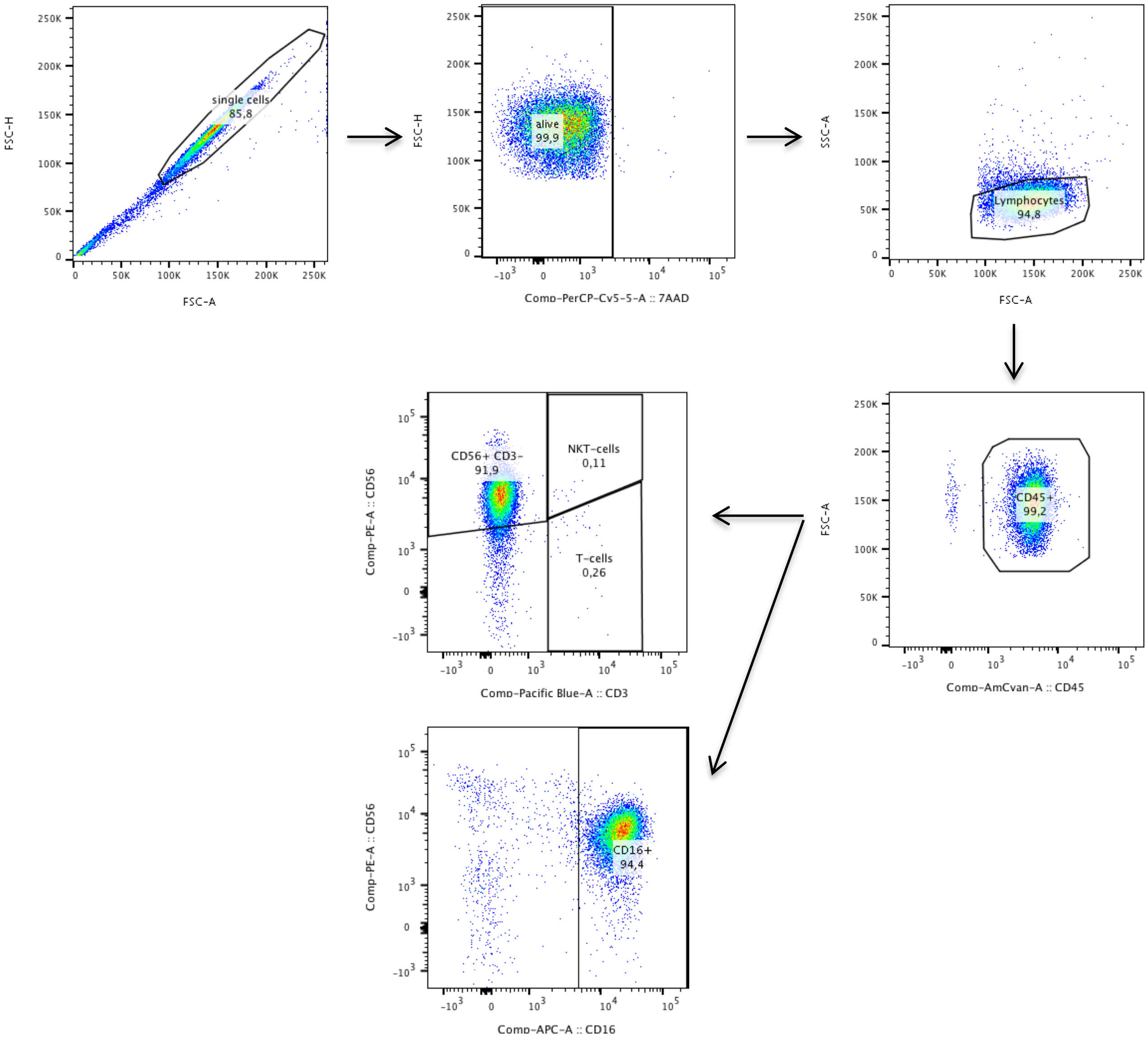

B

Donor	Blood group	Gender	CD56+ CD3- day 0	CD56+ CD3- day 14
#1	A+	M	91.9%	97.8%
#2	AB+	F	84.3%	98.6%
#3	0+	M	92.2%	95.4%
#4	A+	M	96.6%	98.6%
#5	A-	F	94.9%	94.2%
#6	B+	M	92.5%	97.9%
#7	0+	F	81.7%	99.6%
#8	B+	F	77.1%	98.5%
#9	0+	M	91.9%	98.9%
#10	A+	F	87.9%	99.0%
#11	0+	M	92.0%	98.1%
#12	0+	M	91.3%	98.6%
#13	0+	M	94.6%	98.8%

C

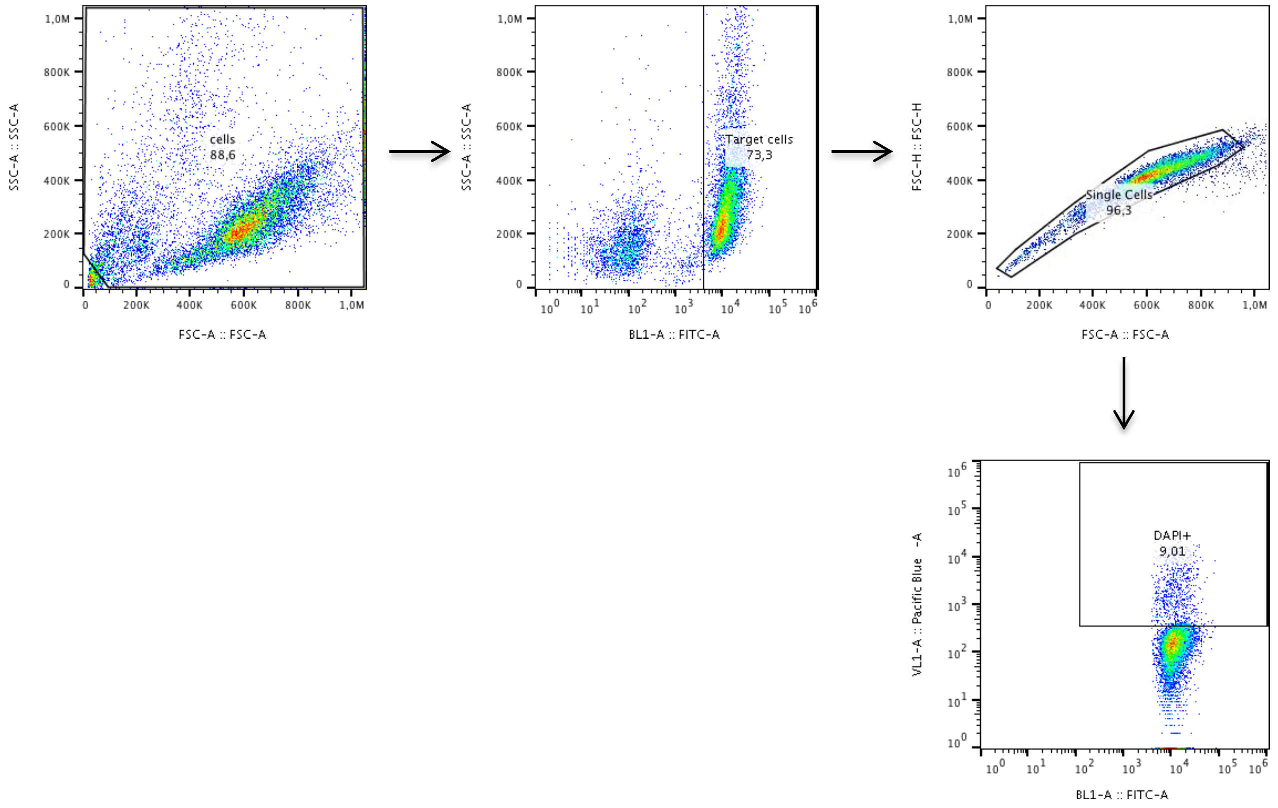

Supplementary Figure 11: NK cell cytotoxicity assays on T-ALL cell lines and dynamic BH3-Profiling after NK cell co-incubation

(A) Repeats of the experiment shown in Figure 5B with NK cells derived from two additional donors. Cell lines were incubated with increasing E:T-ratios (0.2:1, 0.5:1, 1:1 and 3:1) for 24 hours before analysis of cell death by DAPI staining and flow cytometry. (B) RNA-sequencing data of inhibitory and activating ligands for NK cells. T-ALL cell lines were supplied with fresh medium and placed at 37°C and 5% CO_2 for 6 hours. Pellets were collected and RNA was isolated and RNA-sequencing was performed by Novogene GmbH (Munich, Germany); Fragments Per Kilobase of transcript sequence per Millions base pairs sequenced (FMPK), MHC-I receptors is the sum of the expression of *HLA-A, HLA-B, HLA-C, HLA-E, HLA-F*, every line uses its own color gradient. (C) Dynamic BH3-profiling (DBP) of T-ALL cell lines. DBP was performed after 4 hours co-incubation with IL-15-activated NK (E:T-ratio of 0.5:1 for Loucy, ALL-SIL, BE-13 and of 0.05:1 for MOLT-4, CCRF-CEM, Jurkat) cells or controls. Cytochrome c release was analyzed by flow cytometry. Values were normalized to alamethicin as positive control and to DMSO as negative control. Delta-priming was calculated by subtracting normalized cytochrome c release of untreated cells from normalized cytochrome c release of NK cell-treated cells.

A

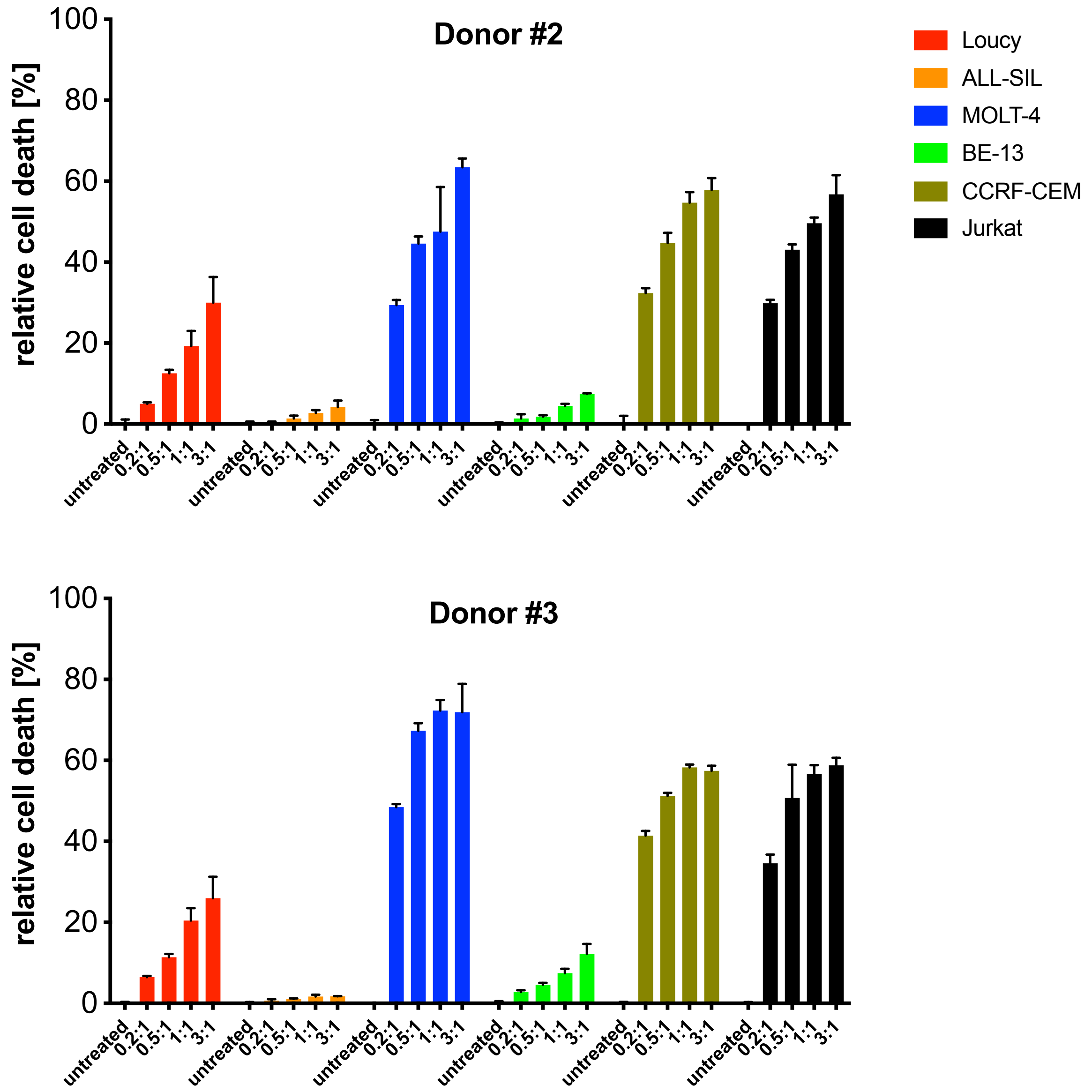

B

Gene name		Gene-FMPK					
		MOLT-4	CCRF-CEM	Jurkat	Loucy	BE-13	ALL-SIL
Death receptors	*TRAILR1*	0.1168	0.1225	0.1539	1.4406	0.0000	0.0000
	TRAILR2	0.1799	2.0493	4.8111	1.5145	0.0000	0.0000
	FAS	3.2486	9.7787	5.2007	2.0992	3.7332	0.6200
Inhibitory receptors	MHC I receptors	46.8495	111.6246	96.6227	506.2537	83.2309	150.3438
	PDL1	0.1872	0.0073	0.3663	0.1488	0.3221	0.0279
	PDL2	0.0000	0.0000	0.0000	0.0000	0.0000	0.0000
	LLT1	0.2020	0.3809	0.0531	0.2101	0.0890	1.8332
	NECTIN3	0.9949	0.2824	0.0505	2.4663	0.1138	2.9568
	NECTIN1	0.0069	2.5340	3.3662	10.3508	1.2392	0.3126
	CEACAM1	0.0192	0.0109	0.0565	0.1058	0.0764	0.0104
	LGALS9	0.4502	6.5089	3.0411	10.6998	4.4193	6.7712
Activating receptors	*MICA*	1.3337	1.0602	2.4990	2.9733	2.0647	1.5135
	MICB	9.8663	7.6955	8.9300	9.4868	11.4094	11.7385
	CD48	2.9159	3.0630	1.1794	1.9153	0.0000	2.2235
	CD70	0.2423	0.1769	0.0371	0.0694	0.0000	0.0187
	RAE1	8.4018	14.9931	7.1771	9.6752	10.9384	12.7807
	ULBP1	1.8399	3.0479	3.6418	0.0000	0.0000	0.0000
	B7H6	4.0285	0.2479	4.7366	1.1556	0.7390	2.7912
	ICAM1	0.2147	0.8451	0.4444	1.5917	0.3494	0.3944

C

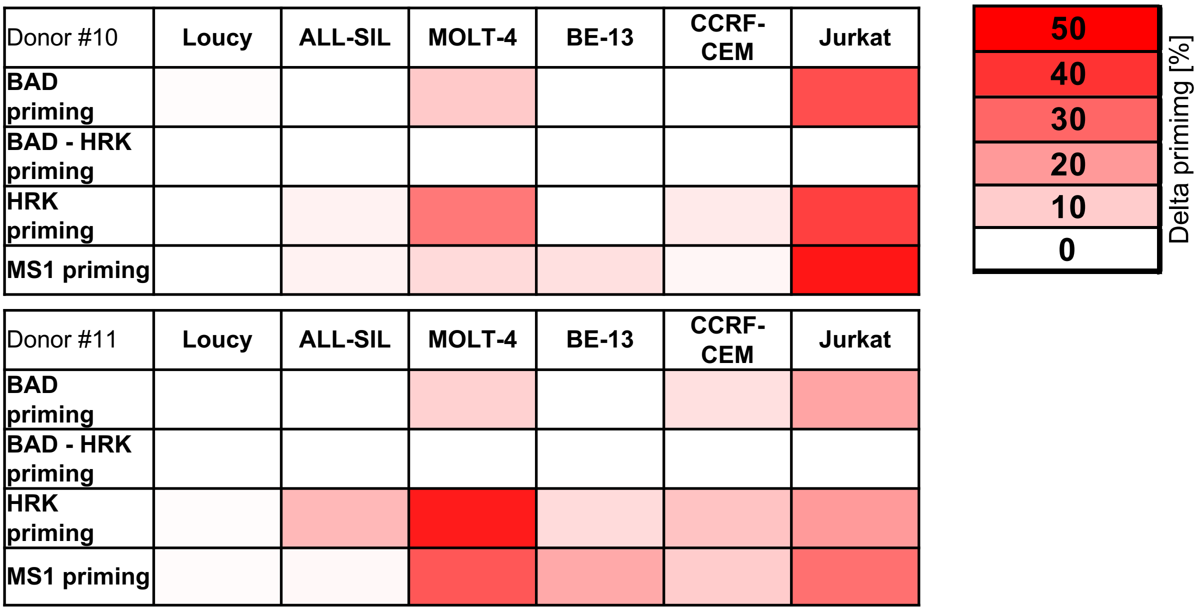

Supplementary Figure 12: Combination of NK cells with AZD4320 using two additional donors

Repetition experiments of Figure 5D using NK cells derived from (A) donor #8 and (B) donor #9. T-ALL cell lines were exposed for 24 hours to increasing concentrations of AZD4320 (1, 10, 50, 100, 250 nM) and/or increasing E:T-ratios of NK cells (0,1:1, 0,2:1, 0,5:1) before analysis of cell death by DAPI staining and flow cytometry. Heatmaps (upper panels) show relative cell death for one of N=3 different donors in triplicates. Efficacy scores (shown above the heatmaps) are calculated as the mean of all normalized cell death rates across the matrix. Interaction landscapes of the combination effects are shown in the respective lower panels. δ-scores were calculated using SynergyFinder. Bliss synergy scores of the whole matrix are shown above and scores of the most synergistic area (MSA) as calculated by synergy finder are shown below the interaction landscapes. Per definition, Bliss synergy scores of less than -10 indicate antagonism, scores between -10 and 10 indicate additive effects and scores above 10 indicate synergism.

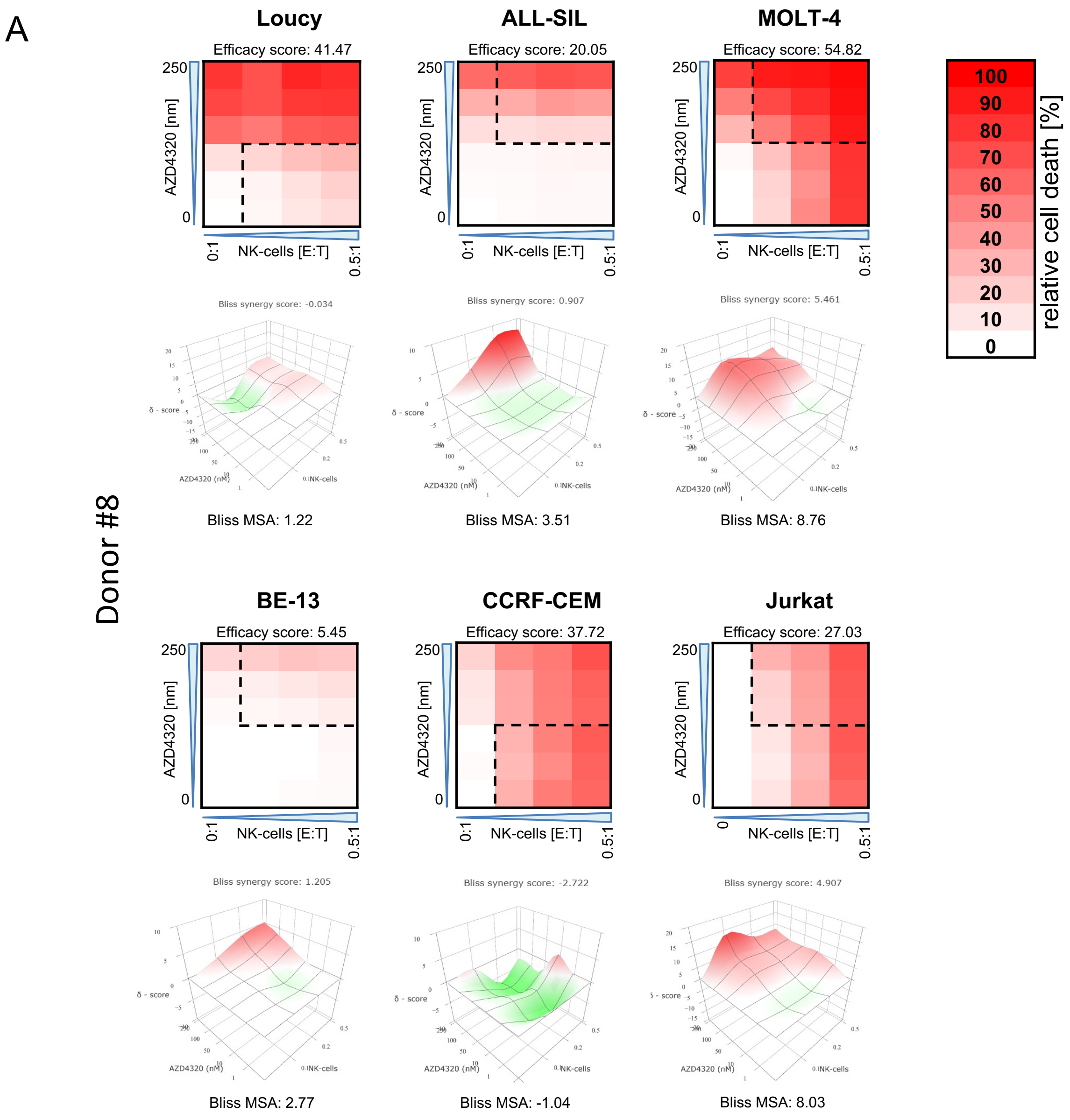

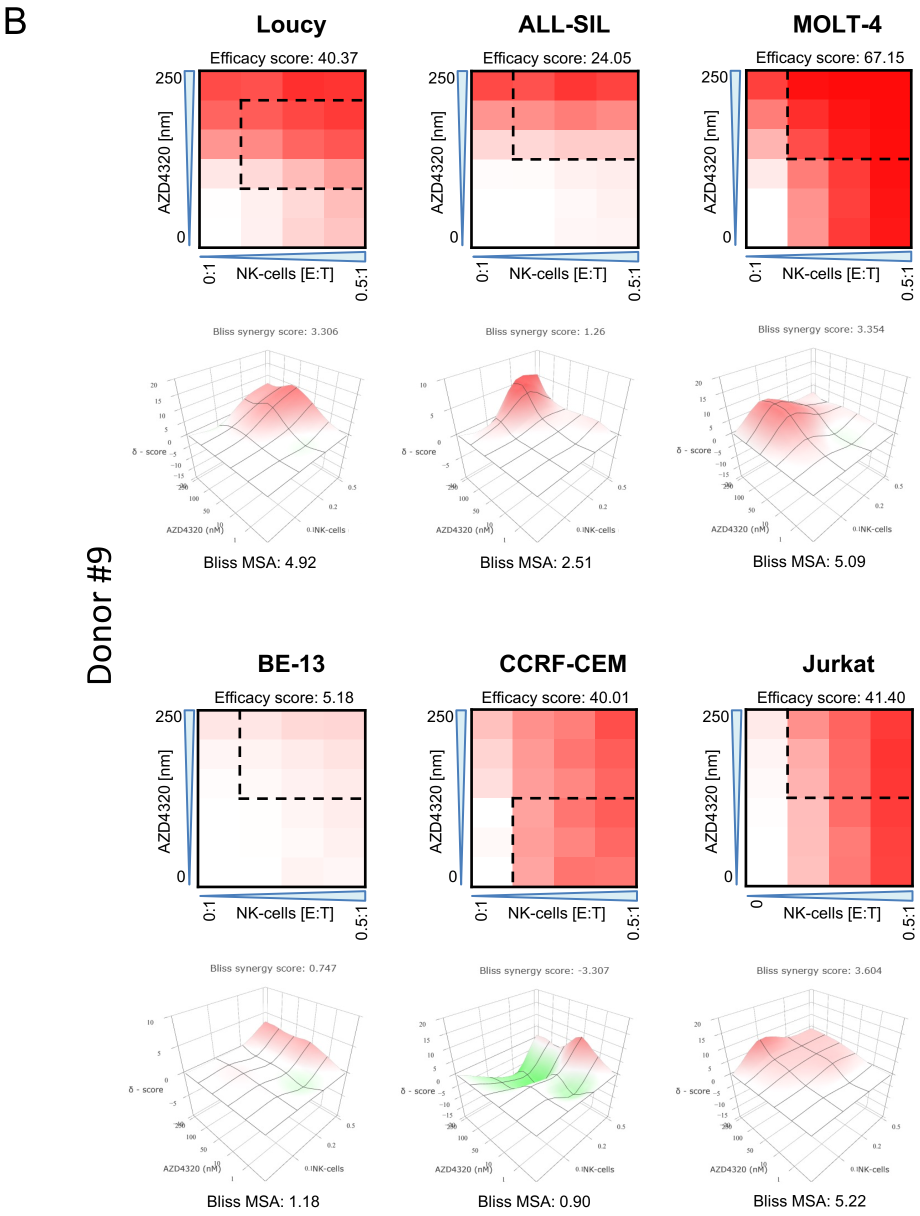
B
Donor #9
Loucy
Efficacy score: 40.37
250
AZD4320 [nm]
0
0:1
NK-cells [E:T]
0.5:1
Bliss synergy score: 3.306
Bliss MSA: 4.92
ALL-SIL
Efficacy score: 24.05
Bliss synergy score: 1.26
Bliss MSA: 2.51
MOLT-4
Efficacy score: 67.15
Bliss synergy score: 3.354
Bliss MSA: 5.09
BE-13
Efficacy score: 5.18
Bliss synergy score: 0.747
Bliss MSA: 1.18
CCRF-CEM
Efficacy score: 40.01
Bliss synergy score: -3.307
Bliss MSA: 0.90
Jurkat
Efficacy score: 41.40
Bliss synergy score: 3.604
Bliss MSA: 5.22

Supplementary Figure 13: Repeat of the combination of NK cells with AZD4320 in PDX samples using additional donors

Repetition experiment of Figure 6B and C using NK cells derived from donor #13. (A) T-ALL PDX were incubated with increasing E:T-ratios (0.1:1, 0.2:1, 0.5:1, 1:1, 2:1 and 3:1) for 24 hours before analysis of cell death by FSC/SSC criteria via flow cytometry. (B) T-ALL PDX were exposed for 24 hours to increasing concentrations of AZD4320 (5, 50, 250, 500, 1000 nM) and/or increasing E:T-ratios of NK cells (0,5:1, 1:1, 2:1) before analysis of cell death by FSC/SSC criteria via flow cytometry. Heatmaps (upper panels) show relative cell death for one of N=2 different donors in triplicates. Efficacy scores (shown above the heatmaps) are calculated as the mean of all normalized cell death rates across the matrix. Interaction landscapes of the combination effects are shown in the respective lower panels. δ-scores were calculated using SynergyFinder. Bliss synergy scores of the whole matrix are shown above and scores of the most synergistic area (MSA) as calculated by synergy finder are shown below the interaction landscapes. Per definition, Bliss synergy scores of less than -10 indicate antagonism, scores between -10 and 10 indicate additive effects and scores above 10 indicate synergism.

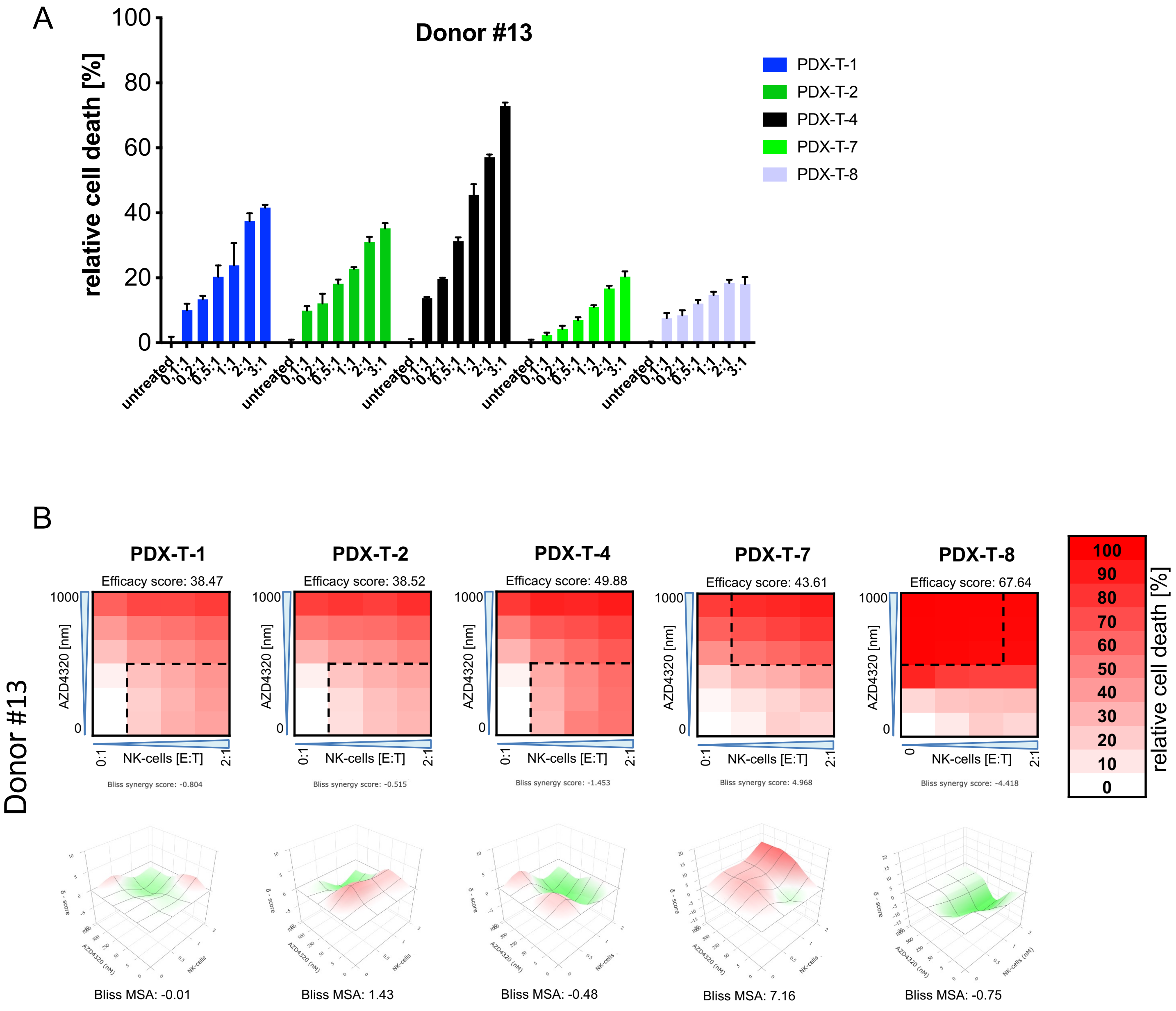

Supplement: Supplementary file 1 — Supplemental material [file 41419_2026_8698_MOESM1_ESM.pdf]
