# Supplementary material for: Therapeutic potential of BH3-mimetics and NK cell-mediated immunotherapy in T-ALL
Source: Cell Death Dis. 2026 Apr 4;17(1):387. doi: 10.1038/s41419-026-08698-x (PMC13068887; doi:10.1038/s41419-026-08698-x)
Supplement: Supplementary file 2 — Original Data [file 41419_2026_8698_MOESM2_ESM.pdf]

Uncropped WBs of Figure 1H

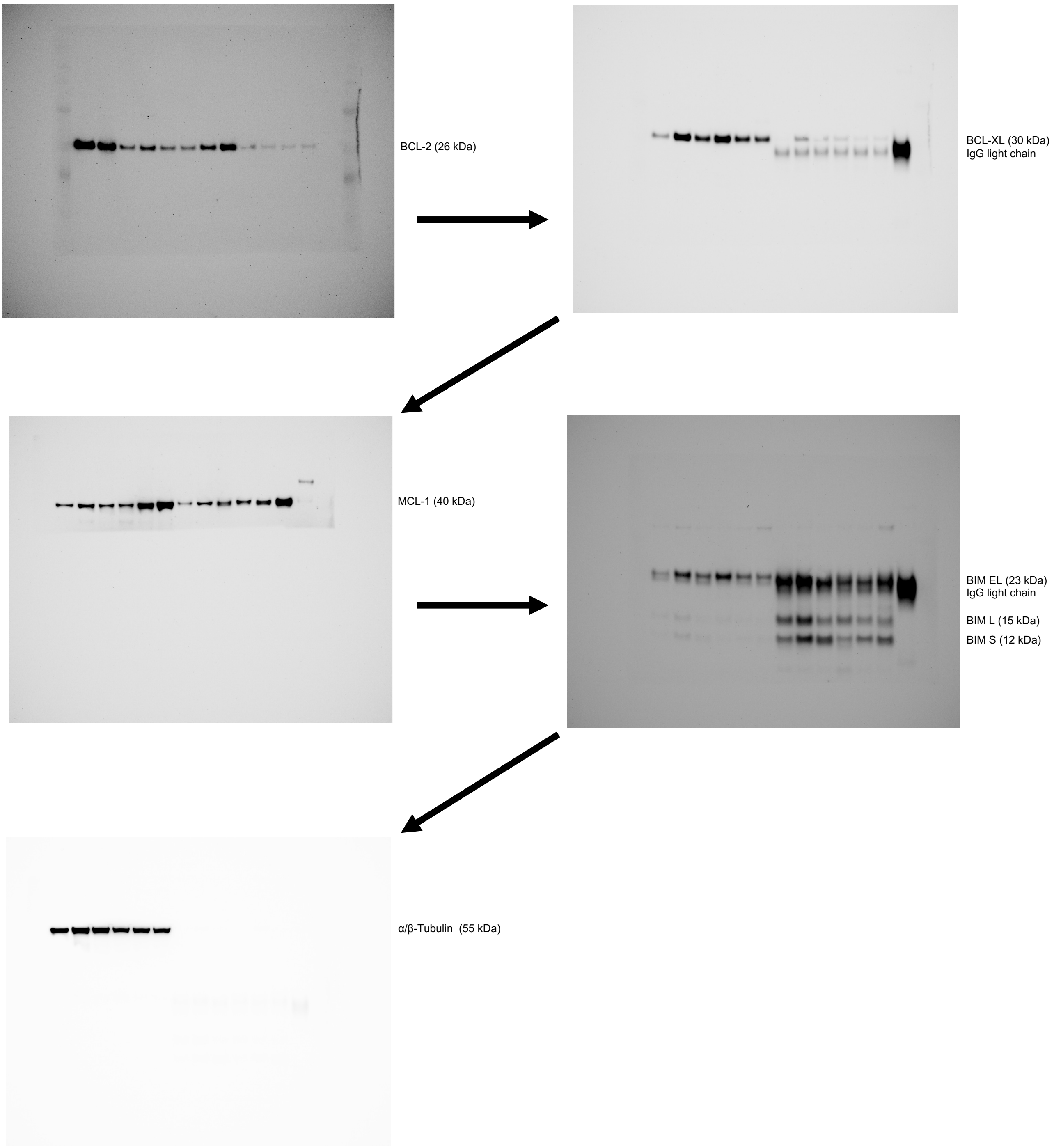

(The Arrows indicate the order in which the primary antibodies were applied)

Uncropped WBs Figure 2C

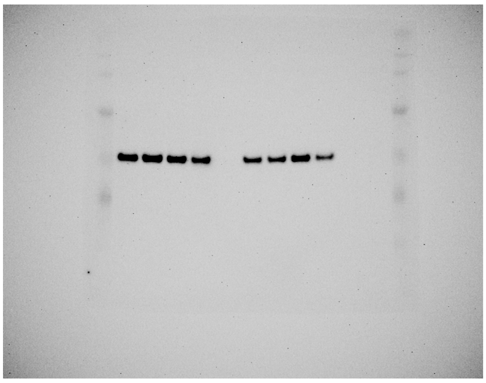

BCL-2 (26 kDa)

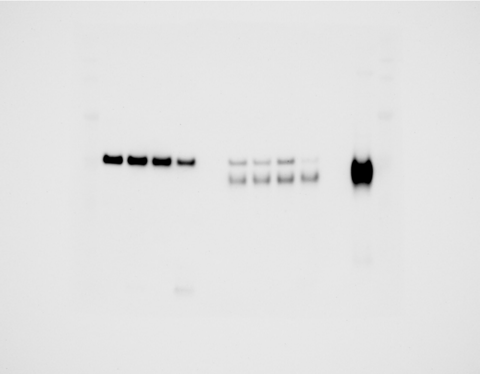

BCL-XL (30 kDa)
IgG light chain

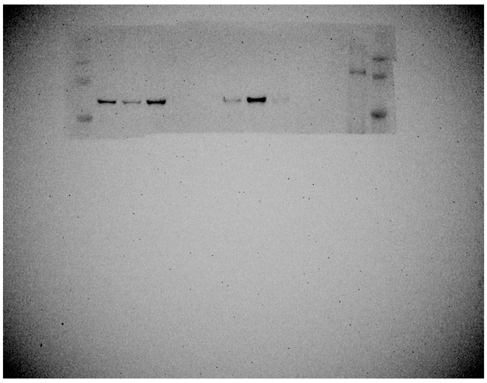

MCL-1 (40 kDa)

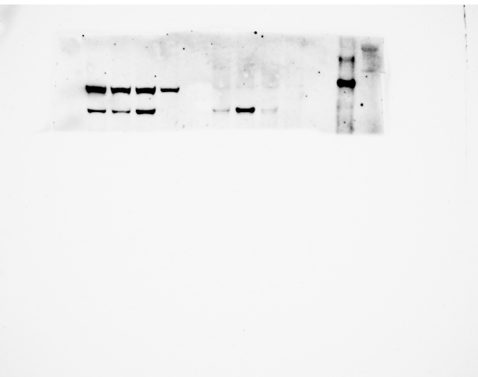

IgG heavy chain
α/β-Tubulin (55 kDa)

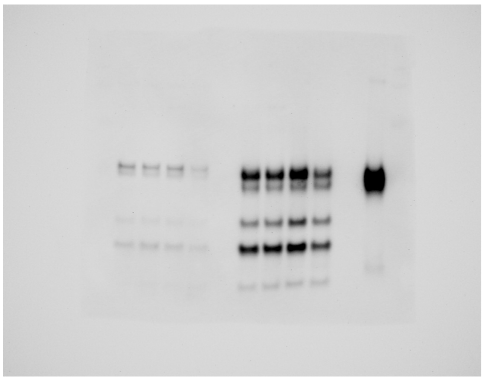

BIM EL (23 kDa)
IgG light chain
BIM L (15 kDa)
BIM S (12 kDa)

(The Arrows indicate the order in which the primary antibodies were applied)

Uncropped WBs of Supplemantary Figure 3A (left)

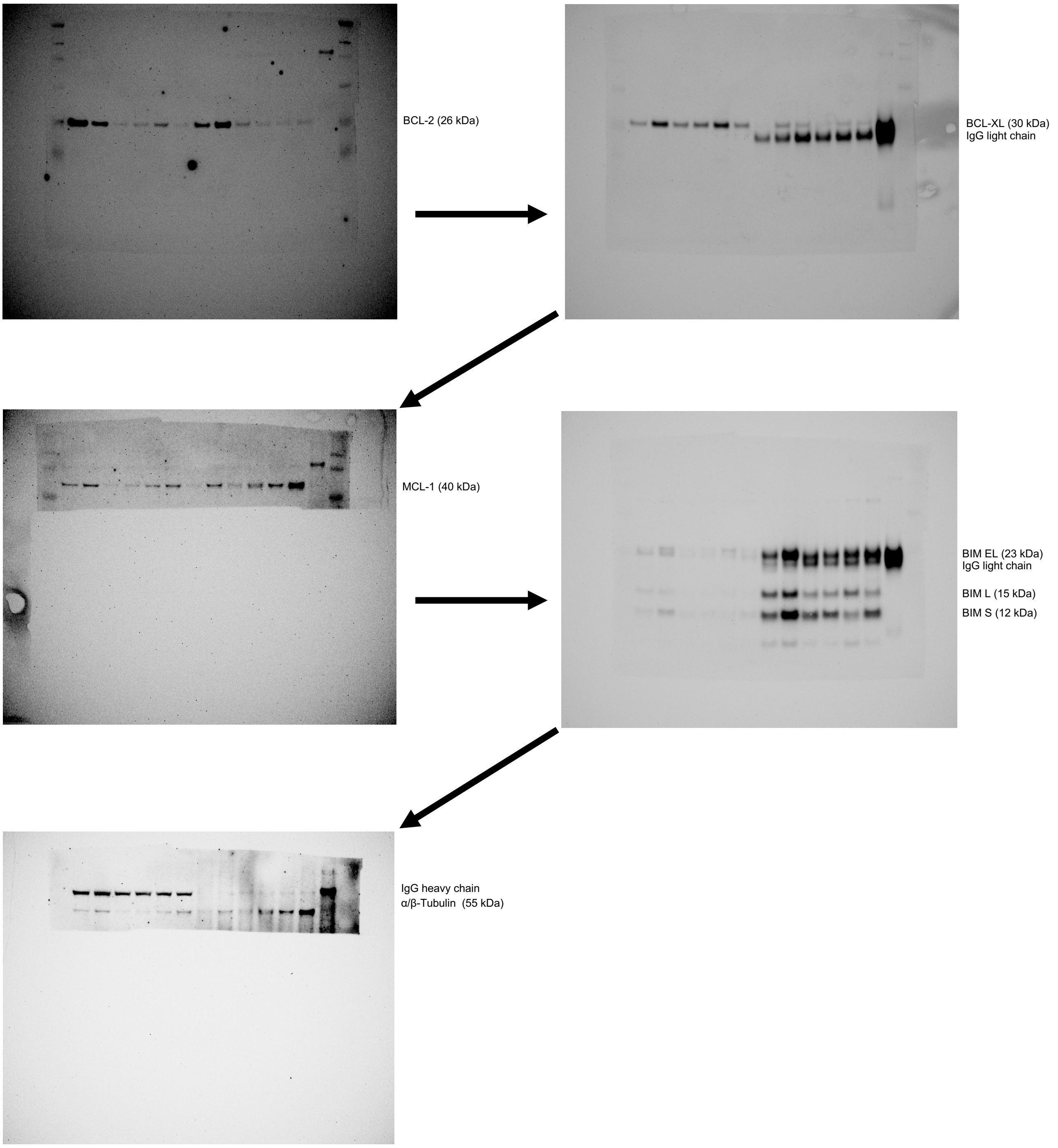

(The Arrows indicate the order in which the primary antibodies were applied)

Uncropped WBs of Supplemantary Figure 3A (right)

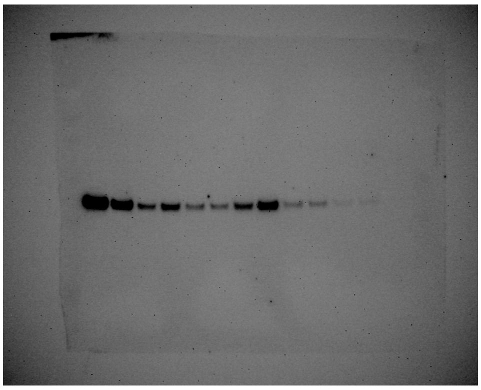

BCL-2 (26 kDa)

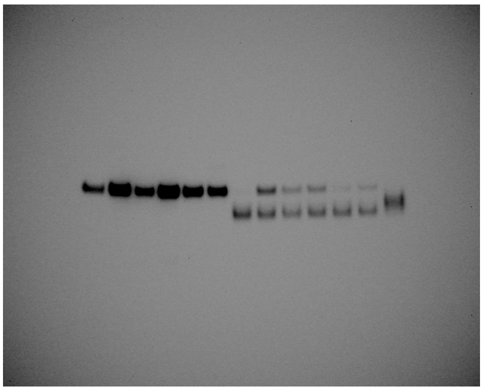

BCL-XL (30 kDa)
IgG light chain

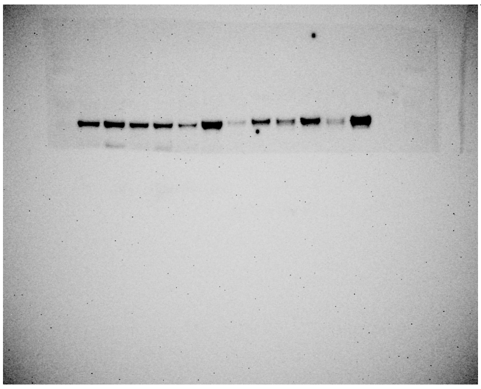

MCL-1 (40 kDa)

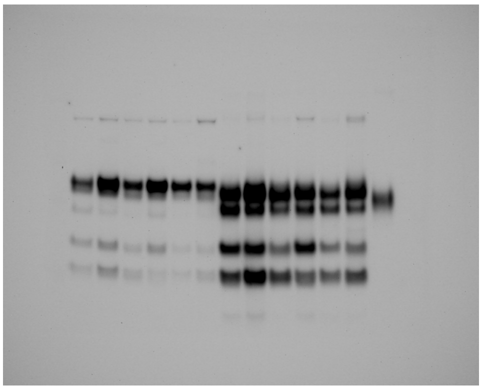

BIM EL (23 kDa)
IgG light chain
BIM L (15 kDa)
BIM S (12 kDa)

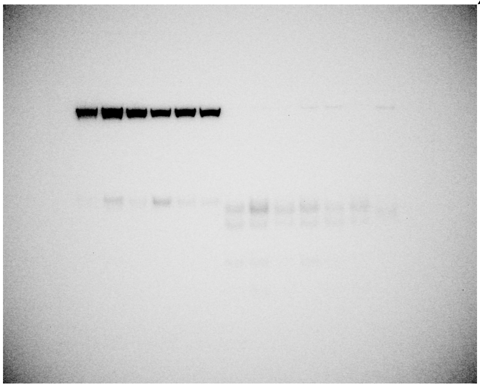

α/β-Tubulin (55 kDa)

(The Arrows indicate the order in which the primary antibodies were applied)

Uncropped WBs Supplementary Figure 3B (left)

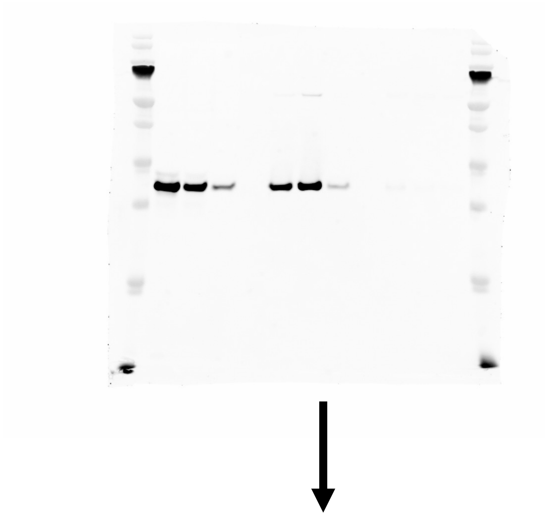

BCL-2 (26 kDa)

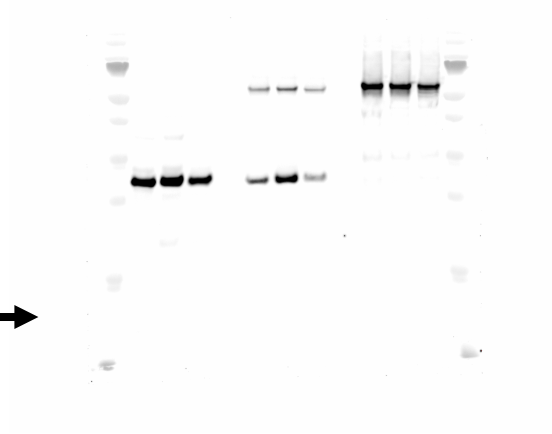

BCL-XL (30 kDa)

(Same WB as igG heavy chain)

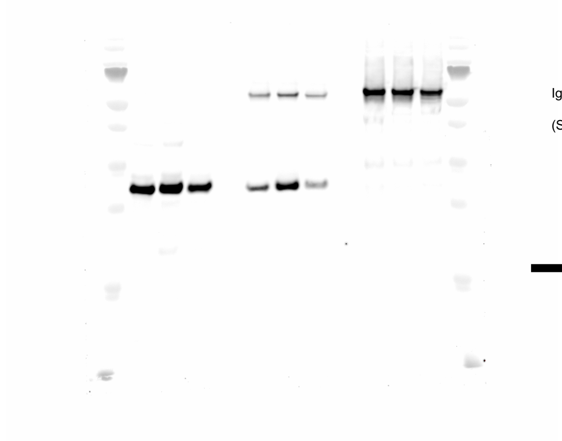

IgG heavy chain (55 kDa)

(Same WB as BCL-XL)

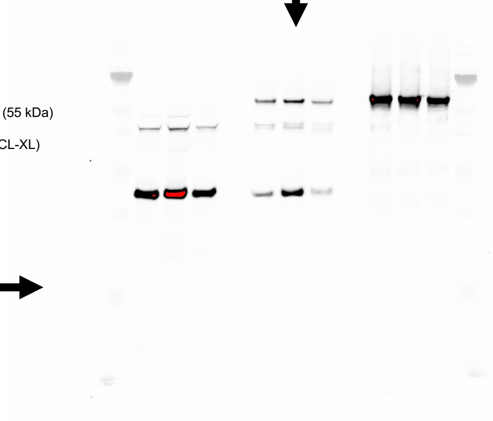

MCL-1 (40 kDa)

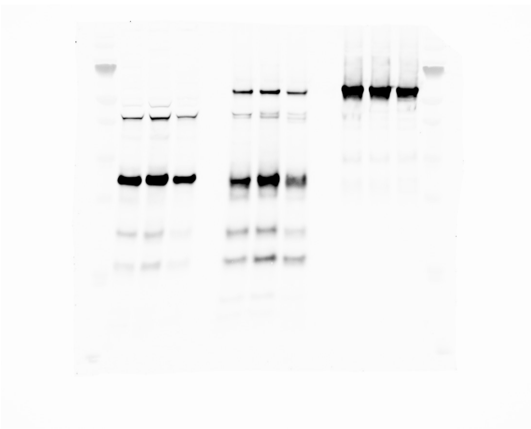

BIM EL (23 kDa)

BIM L (15 kDa)

BIM S (12 kDa)

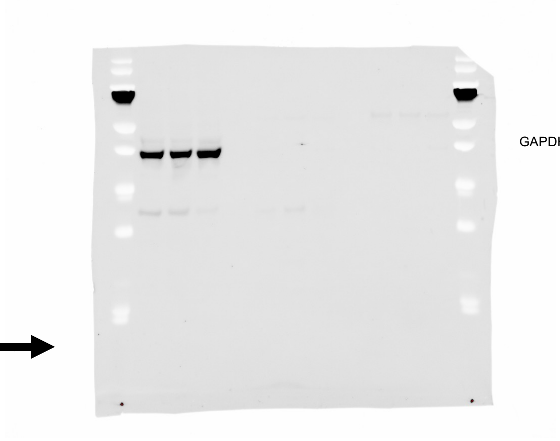

GAPDH (36 kDa)

(The Arrows indicate the order in which the primary antibodies were applied)

Uncropped WBs Supplementary Figure 3B (right)

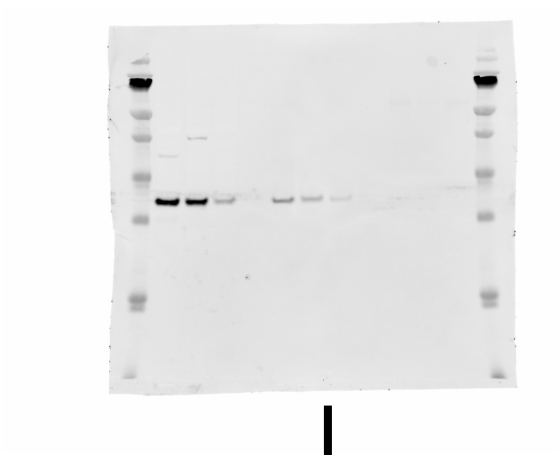

BCL-2 (26 kDa)

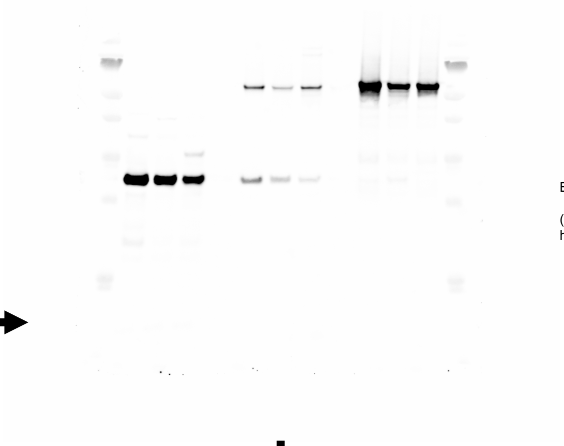

BCL-XL (30 kDa)

(Same WB as igG heavy chain)

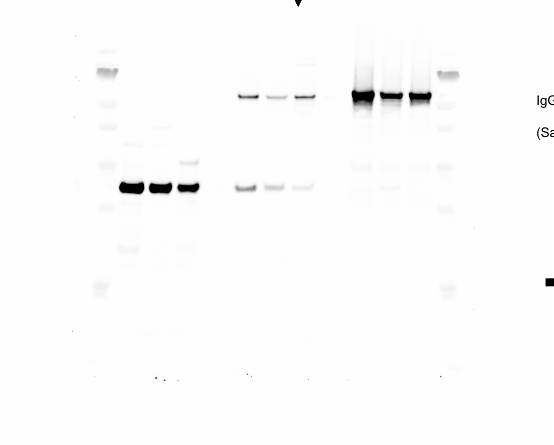

IgG heavy chain (55 kDa)

(Same WB as BCL-XL)

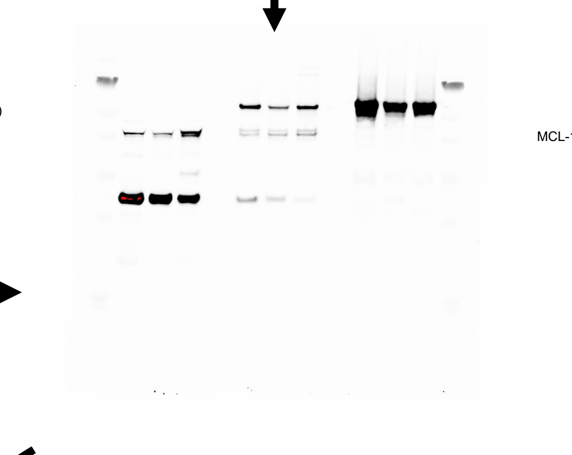

MCL-1 (40 kDa)

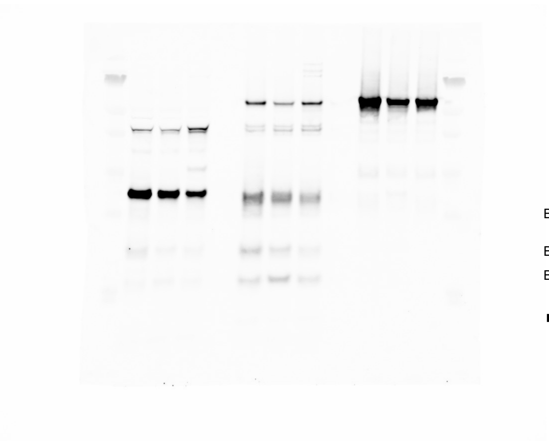

BIM EL (23 kDa)

BIM L (15 kDa)

BIM S (12 kDa)

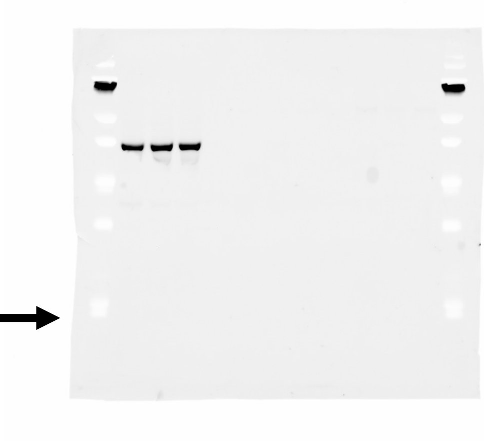

GAPDH (36 kDa)

(The Arrows indicate the order in which the primary antibodies were applied)

Uncropped WBs Supplementary Figure 3C (left)

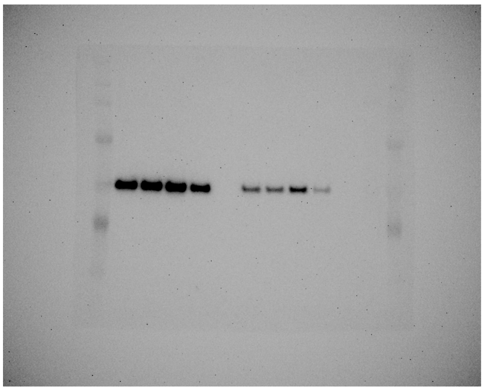

BCL-2 (26 kDa)

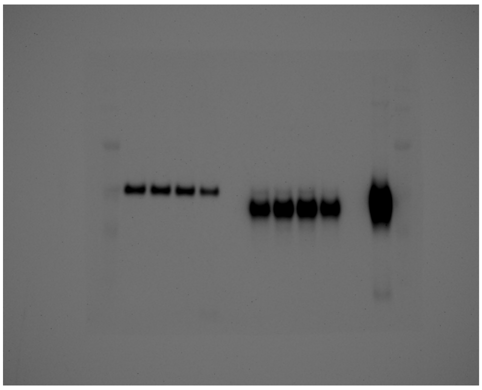

BCL-XL (30 kDa)
IgG light chain

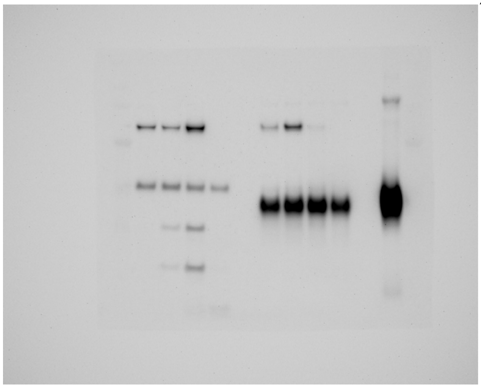

MCL-1 (40 kDa)

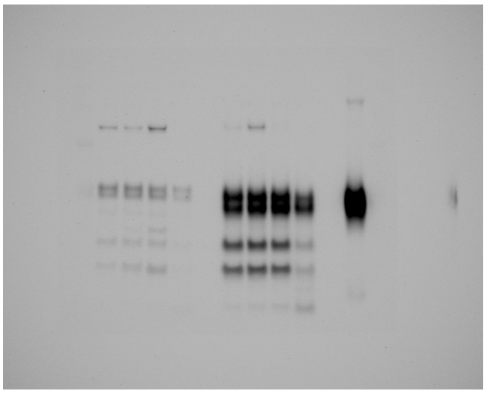

BIM EL (23 kDa)
IgG light chain
BIM L (15 kDa)
BIM S (12 kDa)

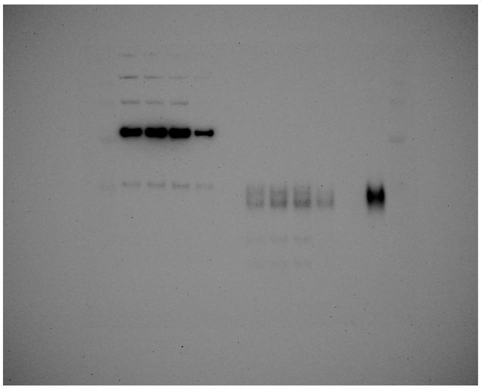

GAPDH (36 kDa)

(The Arrows indicate the order in which the primary antibodies were applied)

Uncropped WBs Supplementary Figure 3C (right)

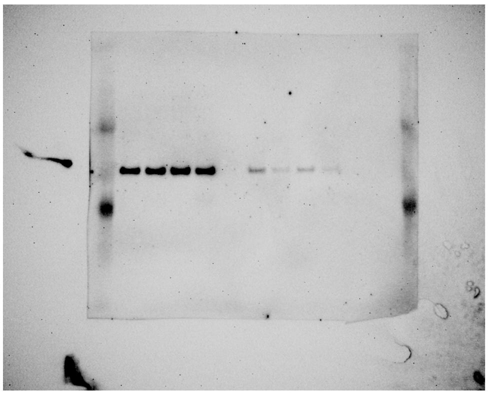

BCL-2 (26 kDa)

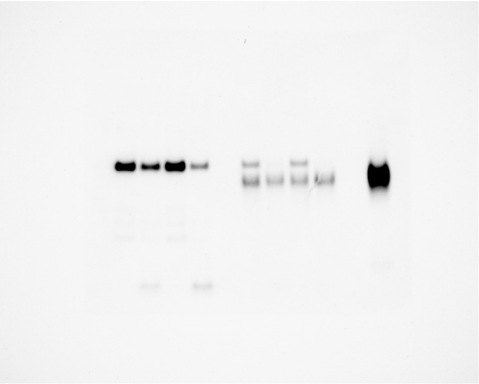

BCL-XL (30 kDa)
IgG light chain

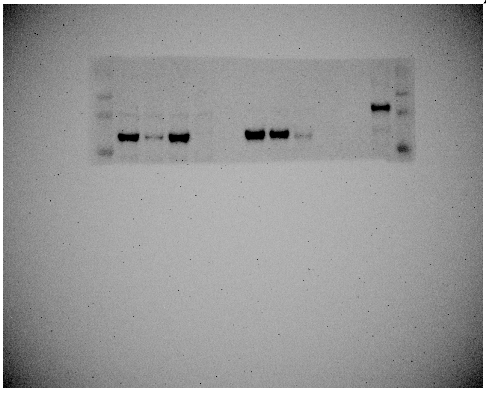

MCL-1 (40 kDa)

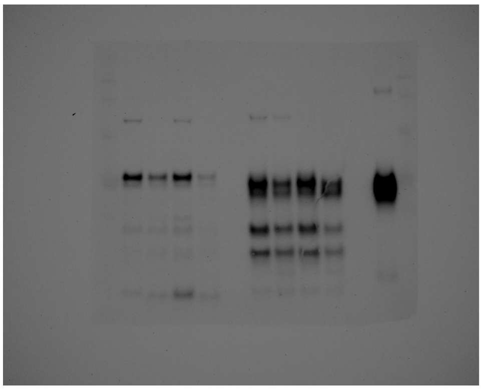

BIM EL (23 kDa)
IgG light chain
BIM L (15 kDa)
BIM S (12 kDa)

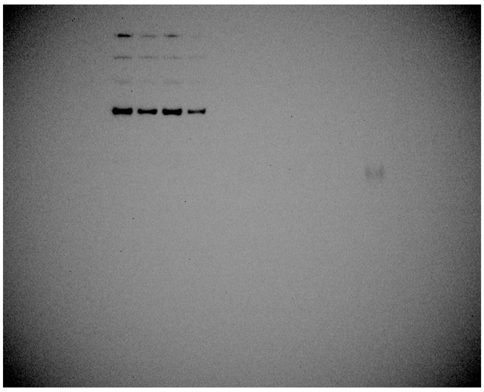

GAPDH (36 kDa)

(The Arrows indicate the order in which the primary antibodies were applied)

Uncropped WBs Supplementary Figure 6A

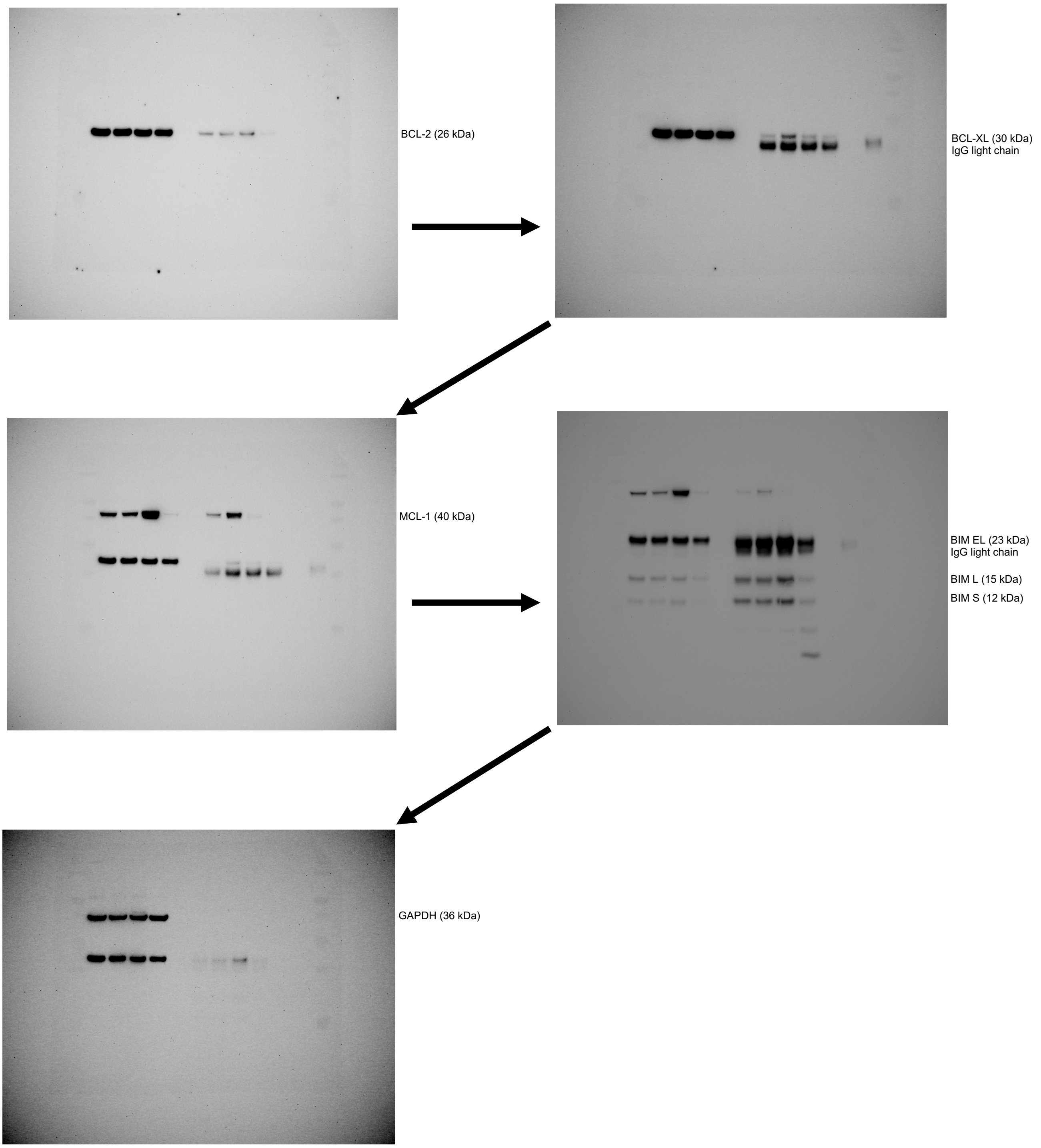

(The Arrows indicate the order in which the primary antibodies were applied)

Uncropped WBs Supplementary Figure 6B

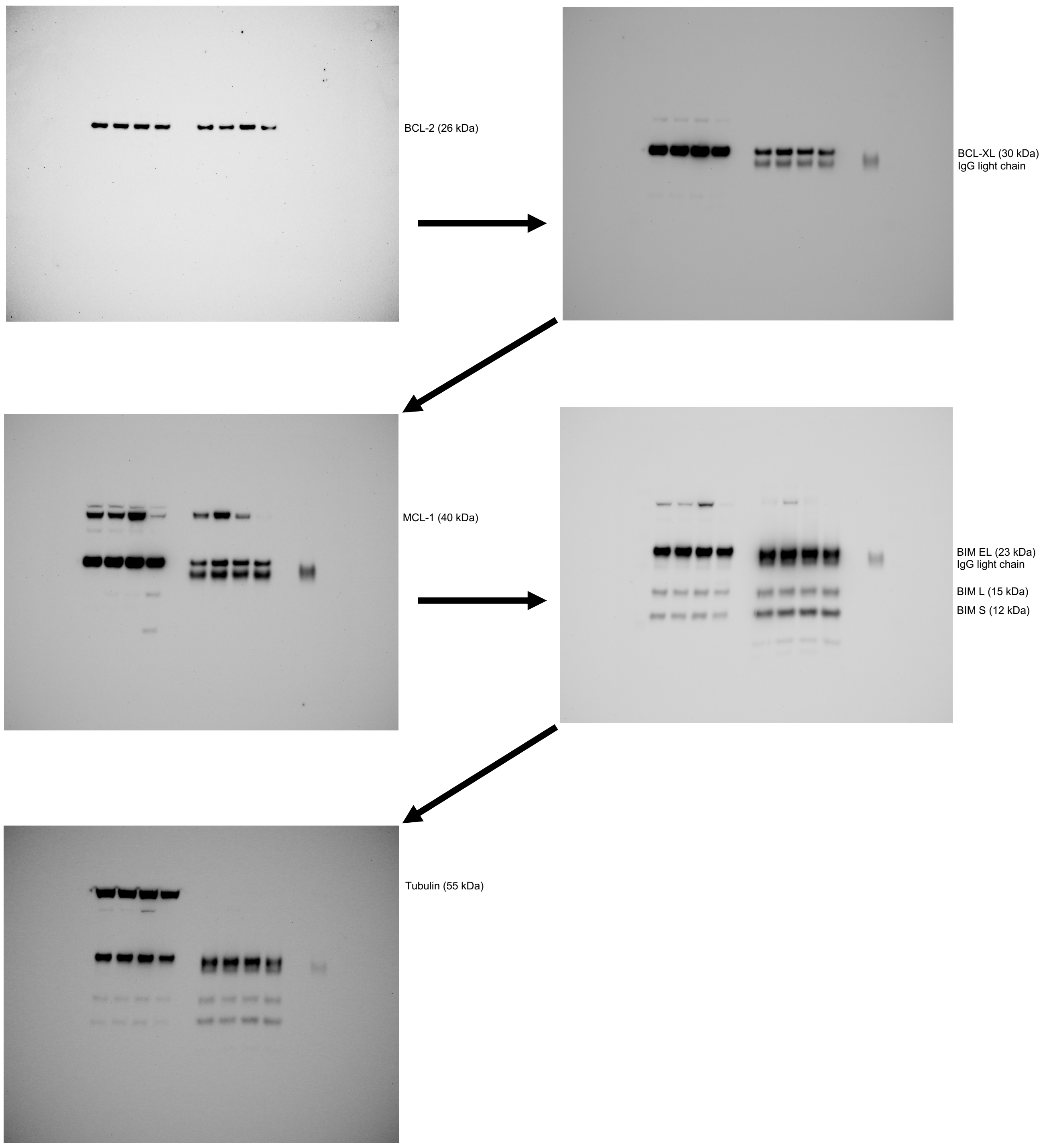

(The Arrows indicate the order in which the primary antibodies were applied)

Uncropped WBs Supplementary Figure 6C

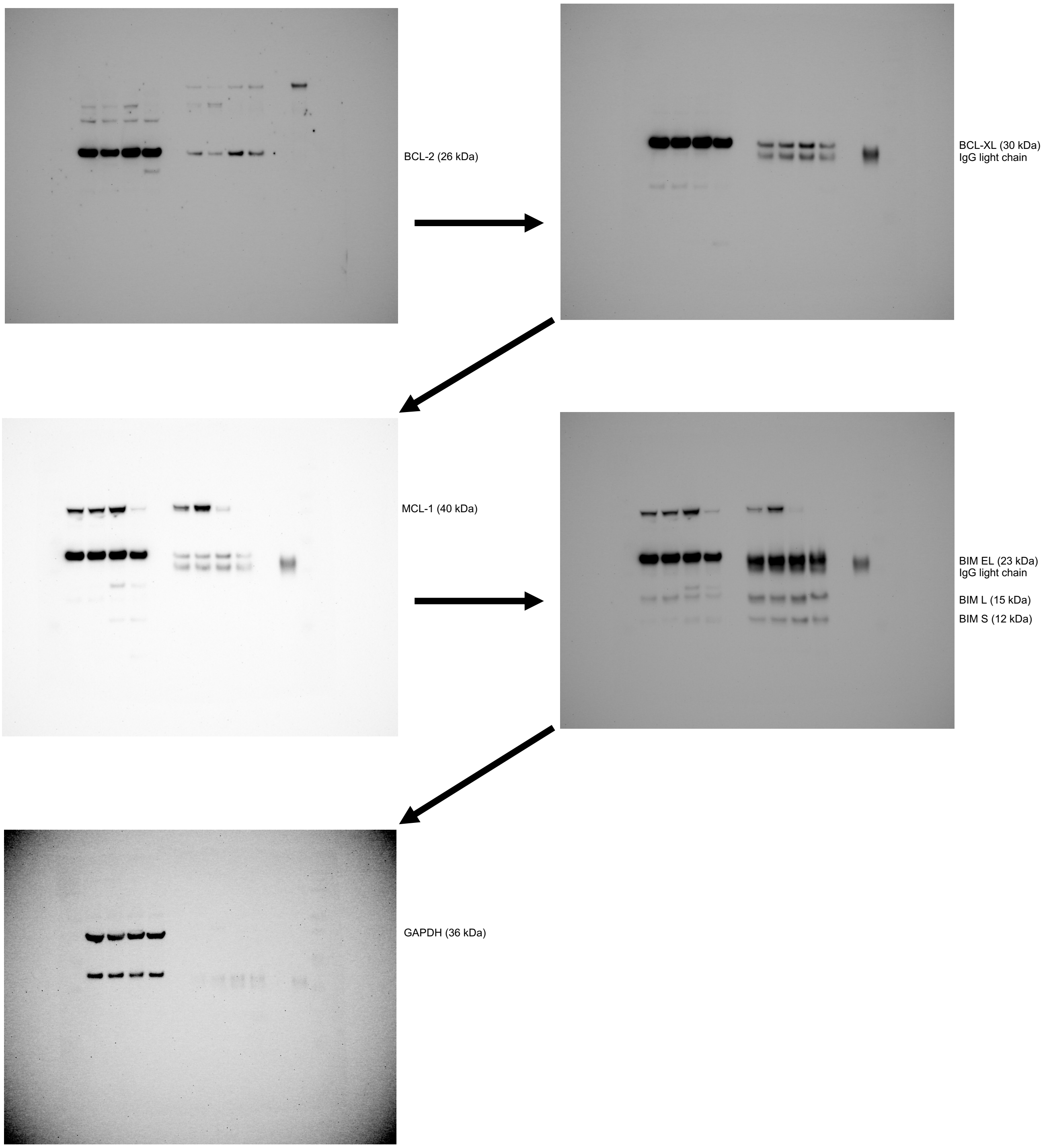

(The Arrows indicate the order in which the primary antibodies were applied)
